# Efficacy comparison of different external traditional Chinese medicine therapies as monotherapy or in combination for premenstrual syndrome: a systematic review and network meta-analysis

**DOI:** 10.3389/fpsyt.2026.1720232

**Published:** 2026-02-04

**Authors:** Yuhong Xie, Jinying Zhao, Shuai Fan, Ning Yang, Huiying Liu, Yuwei Guo, Xu Feng, Ziheng Wang, Min Zhang, Fuchun Wang

**Affiliations:** 1 Department of Acupuncture and Tuina, Changchun University of Chinese Medicine, Changchun, China; 2 Department of Internal Medicine in Traditional Chinese Medicine, Changchun University of Chinese Medicine, Changchun, China; 3 Acupuncture Clinical Center, The Affiliated Hospital to Changchun University of Chinese Medicine, Changchun, China

**Keywords:** acupuncture, ear acupressure, network meta-analysis, premenstrual syndrome, TCM external therapy

## Abstract

**Background:**

Premenstrual syndrome (PMS) is a common gynecological problem that can seriously impair the quality of life of women of childbearing age. Substantial evidence confirms the efficacy of external Traditional Chinese Medicine (TCM) therapies for PMS, though the optimal intervention remains uncertain.

**Objective:**

This study aims to compare the efficacy and safety of external TCM therapies for PMS using Bayesian network meta-analysis, thereby informing evidence-based clinical decisions.

**Methods:**

We systematically searched eight databases for randomized controlled trials (RCTs) evaluating various external TCM therapies for PMS, with all searches conducted through March 10, 2025. The primary outcome measures were overall effective rate and symptom severity scores. We used Stata 17.0 to perform network meta-analysis and compare the therapeutic effects of different interventions on improving PMS symptoms.

**Results:**

The screening process identified 21 eligible RCTs involving 1,818 patients. Most studies demonstrated unclear risk of bias due to insufficient selective reporting details, while six studies were rated high risk for inadequate randomization reporting. The NMA results show that in terms of total effective rate, Jianpi-Shugan acupuncture has the highest SUCRA value (95.7), and for symptom and sign scores, ear acupressure ranks first (SUCRA = 71.2).

**Conclusion:**

External treatment methods of traditional Chinese medicine can serve as a complementary or alternative therapy for PMS; Jianpi-Shugan acupuncture can better enhance the overall effective rate in treating PMS, while ear acupressure is more effective in improving symptom and sign scores. Overall, external treatment methods of TCM for PMS are effective and have almost no side effects, but many high-quality studies are still needed to provide more direct evidence.

**Systematic review registration:**

https://www.crd.york.ac.uk/prospero/, identifier CRD42025643025.

## 1 Introduction

PMS is a clinical syndrome with an incompletely elucidated etiology (1). It is characterized by the cyclic recurrence of psychological symptoms such as anxiety, depression, and mood swings, along with somatic symptoms including edema, breast tenderness, and sleep disturbances during the luteal phase. These symptoms typically subside following the onset of menstruation (2, 3). As one of the most prevalent gynecological issues among women of reproductive age, PMS significantly impairs patients’ quality of life, affecting their physical and psychological well-being, as well as social functioning (4). Epidemiological studies indicate an incidence rate of PMS ranging from 48% to 90% in this population (5). Notably, approximately 3%-10% of PMS patients experience severe emotional dysregulation symptoms; this distinct clinical subtype is defined as Premenstrual Dysphoric Disorder (PMDD) (6). Most PMS patients find it difficult to achieve spontaneous symptom resolution and require clinical intervention (7). Current clinical management of PMS primarily involves pharmacotherapy, including Oral Contraceptive Pills (OCPs), Selective Serotonin Reuptake Inhibitors (SSRIs), and Gonadotropin-Releasing Hormone Agonists (GnRH-a). While these medications can effectively alleviate symptoms (8, 9), their long-term use carries significant risks, such as inducing a low-estrogen state mimicking menopause (pseudo-menopause) and increasing the likelihood of adverse events like gastrointestinal bleeding (10, 11). Consequently, many clinicians observe that their patients frequently seek treatments outside conventional pharmacotherapy to manage PMS symptoms. This includes various Manipulative and Body-Based Practices within Complementary and Alternative Medicine (CAM), such as massage, chiropractic, and acupuncture (12). Thus, external TCM therapies are widely utilized for PMS management. Treatment efficacy can vary due to individual patient constitution, practitioner experience, and treatment duration. Patients may opt for various external TCM interventions, including body acupuncture, moxibustion, and acupoint catgut embedding. These approaches have been proposed as therapeutic options and have demonstrated satisfactory clinical outcomes.

However, previous meta-analyses have primarily investigated the efficacy of acupuncture-based interventions, thereby limiting the scope of their findings. For instance, one meta-analysis on acupuncture for PMS was constrained by its small sample of just 10 randomized clinical trials and narrow assessment range covering only four intervention types. This fails to adequately evaluate the therapeutic efficacy of other TCM external therapies for this condition (13). The meta-analysis conducted by Jiayuan Zhang et al. found that acupuncture provided significant therapeutic efficacy for PMS symptoms in women of reproductive age, with the most frequently used acupoints concentrated on SP6 (Sanyinjiao), LR3 (Taichong), and RN4 (Guanyuan). However, the primary objective of this study was to investigate the timing of the intervention, and it demonstrated no significant difference in outcomes based on when treatment was initiated (14). Neither of the aforementioned studies evaluated the efficacy of a broader range of external TCM therapies for PMS. Given these limitations, there is a compelling need for larger-scale, more comprehensive network meta-analyses (NMAs) to thoroughly investigate the effectiveness of external TCM interventions for PMS. Among all possible TCM external therapy options, a better understanding of the relative effectiveness of active therapeutic interventions holds significant implications for clinical practice and rational resource allocation. This study aims to compare the effects of different external TCM therapies on PMS, thereby providing evidence-based guidance for developing clinical intervention protocols.

## 2 Methods

### 2.1 Search strategy and selection criteria

The systematic review and network meta-analysis protocols have been registered in PROSPERO (registration number: CRD42025643025) and are reported in accordance with the Preferred Reporting Items for Systematic Reviews and Meta-analyses (PRISMA-NMA) guidelines (15).

Two independent reviewers (YHX and MZ) searched PubMed, EMBASE, Cochrane Library, Web of science, Chinese National Knowledge Infrastructure (CNKI), VIP, Wanfang, and China Biomedical Medicine Database (CBM) from the inception of each database until March 10, 2025. We subsequently updated the name on February 6, 2025. Updated data on May 25, 2025, to correct minor errors in keywords, author emails, and application names, and to expand the time of inclusion in the literature. Minor errors corrected on July 24, 2025. The keywords for the literature search were “premenstrual syndrome”, “electroacupuncture”, “acupuncture”, “auricular acupuncture”, “scalp stimulation” and “acupoint injection” in any language in **Supplementary Methods 1**. Two reviewers (YHX and MZ) conducted the systematic literature search independently. Discrepancies were resolved through consensus between the reviewers or arbitration by a third reviewer (FCW). Studies were eligible for inclusion which required meeting all following criteria: 1) Study type: published RCTs of external Chinese medicine treatments for premenstrual syndrome, all of which were two-arm or three-arm trials. Language is limited to Chinese or English. 2) Study population: patients with PMS, all female, of any duration and age. Meet the diagnostic criteria of PMS or PMDD, and the Chinese medicine symptoms of PMS are not limited. 3) Interventions: The interventions in the test group were one or a combination of one or two of the external Chinese medical treatments such as acupuncture, warm acupuncture, electroacupuncture, needle knife, Tuina, moxibustion, cupping and other treatments, with no restrictions on the materials used for treatment, treatment sites, or treatment time. The control group received conventional medication, sham acupuncture, disease health education, or a blank control, or any of the above acupuncture-related therapies. 4) Outcome Measures: Primary endpoint: post-treatment overall effective rate; secondary outcome indicator: symptom and sign scores. Exclusion criteria: 1) Non-RCTs; 2) Involves combining other therapeutic measures, such as Western medicine drugs, physical therapy, rehabilitation, and exercise; 3) missing data and outcome indicators; 4) duplicate publication, not found the original text, etc; 5) reviews, animal experiments, treatment summaries, results, conference and case reports.

After importing all literature into EndNote 21 and removing duplicates, dual reviewers (JYZ and YWG) independently screened titles and abstracts for determining the study relevant. Eligible studies underwent full-text retrieval and assessment by two independent assessors. Data extraction was similarly performed independently. Discrepancies emerging during these procedures were resolved through consensus adjudication or third-party consultation (ZHW) when indicated.

The extracted data includes: 1) author; 2) publication year; 3) Participant characteristics: age and sample size; 4) Intervention parameters: type, individual session duration, administration frequency, and total treatment time; 5) Outcome measures: overall effective rate and symptom severity scores. For data reported in the same randomized controlled trial from multiple sources or with different follow-up periods, immediate results after intervention are selected as the experimental outcome. If the data is missing, we will contact the author to request the original data. Our research focuses on baseline and post intervention data. (see **Supplementary Table S1**) We extracted the mean and standard deviation (SD) of baseline changes, which are the main data sources analyzed in this article. If the article does not provide standard deviation, standard error, 95% confidence interval (CI), range, and quartile are used for calculation. The formula used for calculation can be found in **Supplementary Method 2**. The extracted data is stored in a pre-created structured data table.

### 2.2 Data analysis

First, a paired meta-analysis was performed using STATA 17.0. For binary variables, the relative risk (RR) and 95% CI were used for analysis, while the mean difference (MD) was applied for continuous variables. Cochran's I-square statistic (I²) was utilized to determine heterogeneity. A fixed-effects model was selected for data analysis when I² < 50%, and a random-effects model was chosen when I² > 50%. For both RR and MD, a p-value < 0.05 was considered to indicate statistical significance.

We performed a network meta-analysis using Stata 17.0 to compare the efficacy of different external Traditional Chinese Medicine therapies for premenstrual syndrome. To account for potential heterogeneity between studies, a random-effects model was specified in advance. Binary outcomes were pooled and analyzed using risk ratios as the effect measure, while continuous outcomes were analyzed using mean differences; both are reported with their 95% confidence intervals. Results were considered statistically significant if the 95% confidence interval did not include the null value. Network graphs generated through Stata’s mvmeta package visualized interventions as proportionally-sized nodes reflecting sample magnitude, interconnected by lines whose thickness corresponded to direct comparison sample sizes. The global consistency model was applied when p-values exceeded 0.05, while local inconsistency underwent node-splitting examination and closed loops received loop inconsistency assessment. Relative effects were presented in league tables structured as paired comparison matrices, supplemented by cumulative ranking curves where Surface Under Cumulative Ranking Area values quantified intervention efficacy, and higher values indicating superior effects. Publication bias evaluation and small sample effects employed comparative corrected funnel plots created in Stata. A funnel plot is a graphical tool used in meta-analysis to assess the potential for publication bias or small-study effects visually. In this plot, the effect estimates from individual studies are plotted against a measure of their precision, typically the inverse of the standard error. The red line represents the null hypothesis, which means that there is no difference between the effect size of each study and the estimated comprehensive effect of the corresponding comparison. The green line is the regression line.

We used the Cochrane bias risk tool ROB2 to assess bias risk in trials (16). Therefore, we evaluated six types of bias risks, including Randomization process, Deviations from intended interventions, Missing outcome data, Measurement of the outcome, Selection of the reported result, Overall Bias. The risk classification includes low, high, and unclear, independently determined by two investigators (NY and HYL), with disagreements resolved by a third reviewer (YW).

## 3 Results

### 3.1 Study selection

**Figure 1:**
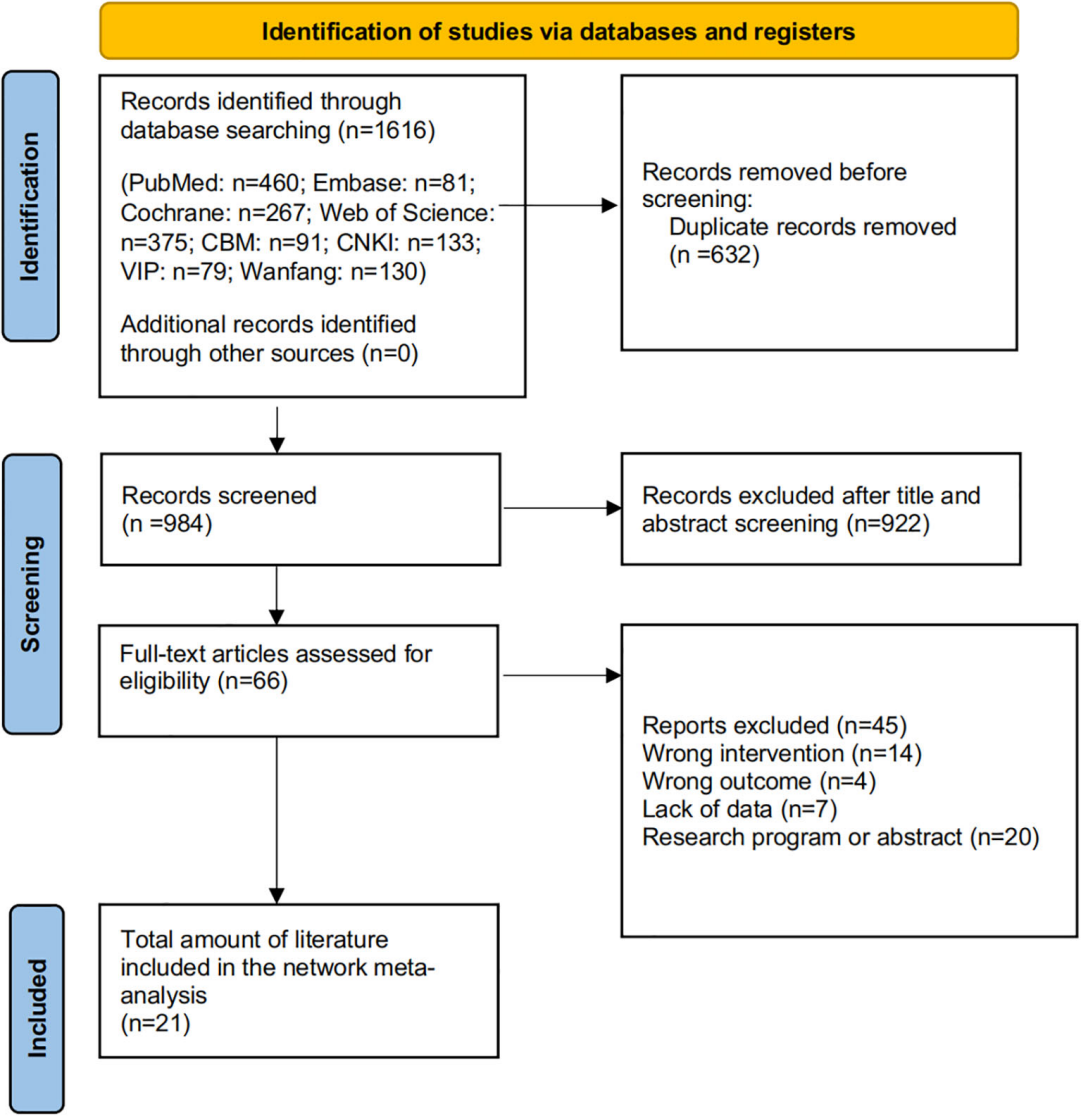
This is the flowchart of the literature selection process for this study, detailing the specific circumstances of each step, ultimately resulting in the inclusion of 21 articles in the research.

Database searches identified a total of 1,616 records. Following the removal of duplicates, 984 unique records were screened. After reviewing titles and abstracts, 922 records were excluded, leaving 66 full-text articles for detailed assessment. Of the 45 articles excluded, the reasons for exclusion were as follows: 20 were study protocols or abstracts only, 14 utilized ineligible interventions, 7 had incomplete or unavailable data, and 4 reported irrelevant outcome measures. Ultimately, 21 studies (see **Supplementary Methods 4**) involving 1,818 participants were included in the network meta-analysis (**Figure 1** and **Supplementary Figure S1**).

### 3.2 Risk of bias evaluation

**Figure 2:**
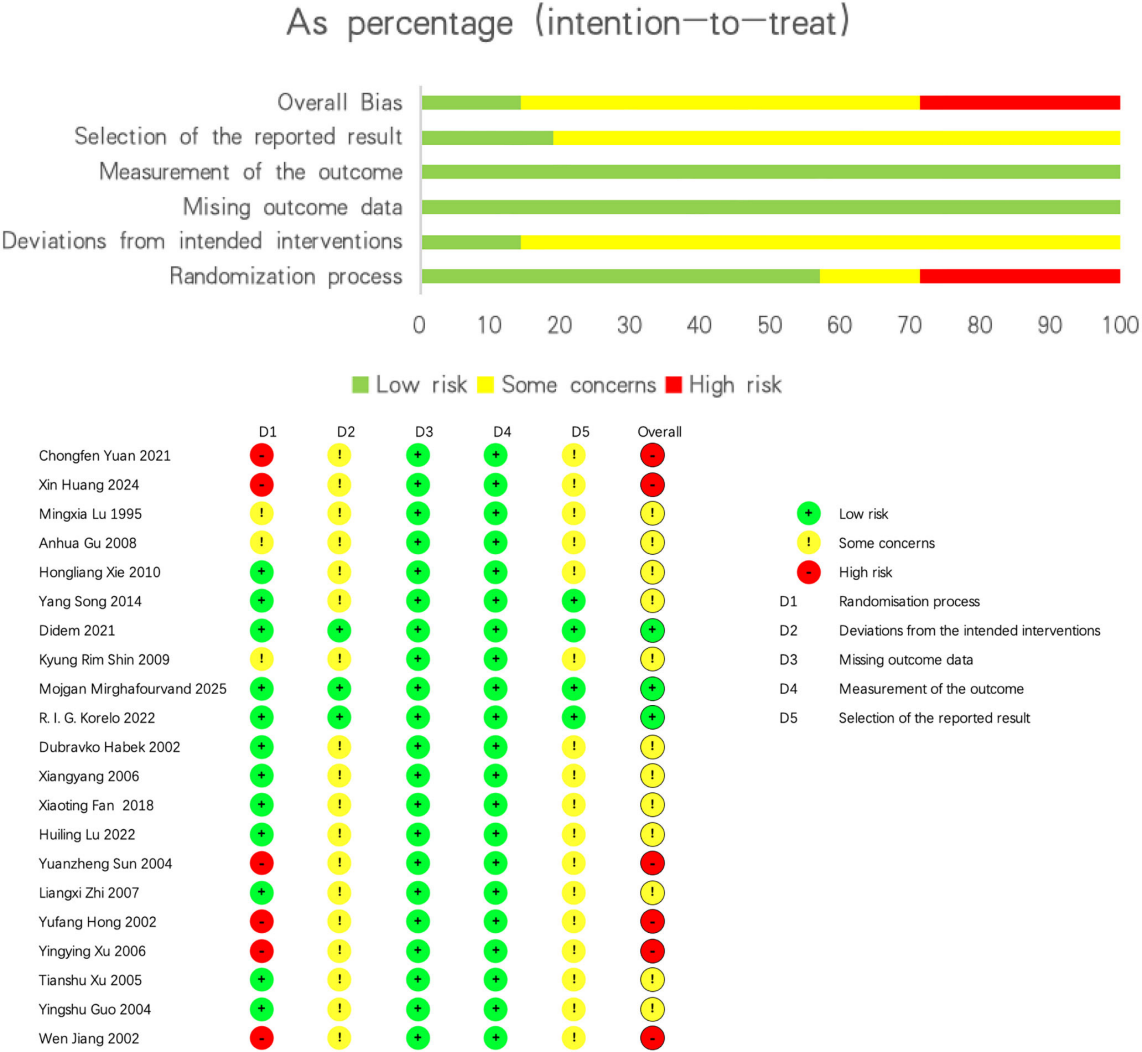
Study quality assessment.

**Figure 2** presents the risk of bias assessment for the included studies. Overall, three studies were judged to have a low risk of bias, twelve studies an unclear risk of bias, and six studies a high risk of bias.

### 3.3 Pairwise meta-analysis

We conducted a meta-analysis of 13 pairs to compare the overall effective rate of different traditional Chinese medicine external treatment methods on premenstrual syndrome (**Table 1**). Compared with Western medicine, acupuncture (3 RCTs, RR = 1.45, 95%CI=1.23–1.72, I²=0.0%, p=0.000), ‘8’ shaped ring acupuncture method (1 RCT, RR = 1.64, 95%CI=1.16–2.33, p=0.005), hypodermic catgut embedding (2 RCTs, RR = 1.25, 95%CI=1.09–1.42, I²=0.0%, p=0.001), ear acupressure (1 RCT, RR = 1.42, 95%CI=1.04–1.93, p=0.027), scalp acupuncture combined with electroacupuncture (1 RCT, RR = 1.42, 95%CI=1.07–1.88, p=0.015), and acupuncture combined with point injection (1 RCT, RR = 1.24, 95%CI=1.03–1.48, p=0.022), were more effective in improving the total effective rate of PMS; compared with placebo control group, acupuncture (1 RCT, RR = 13.22, 95%CI=1.94–89.96, p=0.008), and ear acupressure (1 RCT, RR = 2.13, 95%CI=1.42–3.17, p=0.000) were more effective; compared with acupuncture, moxibustion (2 RCTs, RR = 0.79, 95%CI=0.69–0.92, I²=0.0%, p=0.002), Linggui-Bafa acupuncture (1 RCT, RR = 0.76, 95%CI=0.61–0.95, p=0.016), and Jianpi-Shugan acupuncture (1 RCT, RR = 0.63, 95%CI=0.44–0.92, p=0.015) were more effective; moreover, acupuncture (1 RCT, RR = 20.95, 95%CI=4.39–100.02, p=0.000) was more effective than blank control group. However, there was no statistically significant difference in ear acupressure combined with moxibustion vs conventional Western medicine group.

**Table 1:** Pairwise meta-analysis of overall effective rate.

Intervention	Number	RR (95%CI)	*I*2 (%)	*P*
MOX vs ACU	2	**0.79 (0.69, 0.92)**	0.0	0.002
LGBF vs ACU	1	**0.76 (0.61, 0.95)**	–	0.016
JPSG vs ACU	1	**0.63 (0.44, 0.92)**	–	0.015
ACU vs BCG	1	**20.95 (4.39, 100.02)**	–	0.000
ACU vs PCG	1	**13.22 (1.94, 89.96)**	–	0.008
EA vs PCG	1	**2.13 (1.42, 3.17)**	–	0.000
ACU vs CWMG	3	**1.45 (1.23, 1.72)**	0.0	0.000
‘8’SRAM vs CWMG	1	**1.64 (1.16, 2.33)**	–	0.005
EA+MOX vs CWMG	1	1.16 (0.97, 1.38)	–	0.093
HCE vs CWMG	2	**1.25 (1.09, 1.42)**	0.0	0.001
EA vs CWMG	1	**1.42 (1.04, 1.93)**	–	0.027
SA+ETA vs CWMG	1	**1.42 (1.07, 1.88)**	–	0.015
ACU+PI vs CWMG	1	**1.24 (1.03, 1.48)**	–	0.022

The bold font indicates a statistical difference. EA, Ear Acupressure; MOX, Moxibustion; PCG, Placebo Control Group; ‘8’SRAM, ‘8’Shaped Ring Acupuncture Method; EA+MOX, Ear Acupressure Combined with Moxibustion; HCE, Hypodermic Catgut Embedding; ACU, Acupuncture; SA+ETA, Scalp Acupuncture Combined with Electroacupuncture; JPSG, Jianpi-Shugan Acupuncture; LGBF, Linggui-Bafa Acupuncture; ACU+PI, Acupuncture Combined with Point Injection; CWMG, Conventional Western Medicine Group; BCG, Blank Control Group.

In terms of symptom and sign scores (**Table 2**), acupuncture (2 RCTs, MD=-8.24, 95%CI=-16.29– -0.18, I²=69.7%, p=0.045), moxibustion (1 RCT, MD=-14.36, 95%CI=-21.11– -7.61, p=0.000), acupressure (2 RCTs, MD=-13.67, 95%CI=-26.22– -1.13, I²=77.5%, p=0.033), and auricular microneedle (1 RCT, MD=-7.10, 95%CI=-7.86– -6.34, p=0.000) were more effective than blank control group; acupuncture (1 RCT, MD=-4.82, 95%CI=-6.56– -3.08, p=0.000), ‘8’shaped ring acupuncture method (1 RCT, MD=-7.10, 95%CI=-9.89– -4.32, p=0.000), ear acupressure combined with moxibustion (1 RCT, MD=-7.00, 95%CI=-8.78– -5.22, p=0.000), hypodermic catgut embedding (1 RCT, MD=-5.26, 95%CI=-6.75– -3.77, p=0.000), and ear acupressure (1 RCT, MD=-10.70, 95%CI=-15.77– -5.63, p=0.000) were more effective than conventional Western medicine group; in addition, ear acupressure (1 RCT, MD=-2.36, 95%CI=-3.43– -1.29, p=0.000) was more effective than placebo control group. There was no statistically significant difference when comparing acupuncture with moxibustion.

**Table 2:** Pairwise meta-analysis of symptom and sign scores.

Intervention	Number	MD (95%CI)	*I*2 (%)	*P*
ACU vs BCG	2	**-8.24 (-16.29, -0.18)**	69.7	0.045
MOX vs BCG	1	**-14.36 (-21.11, -7.61)**	–	0.000
APR vs BCG	2	**-13.67 (-26.22, -1.13)**	77.5	0.033
AM vs BCG	1	**-7.10 (-7.86, -6.34)**	–	0.000
ACU vs CWMG	1	**-4.82 (-6.56, -3.08)**	–	0.000
‘8’SRAM vs CWMG	1	**-7.10 (-9.89, -4.32)**	–	0.000
EA+MOX vs CWMG	1	**-7.00 (-8.78, -5.22)**	–	0.000
HCE vs CWMG	1	**-5.26 (-6.75, -3.77)**	–	0.000
EA vs CWMG	1	**-10.70 (-15.77, -5.63)**	–	0.000
ACU vs MOX	1	0.56 (-7.68, 8.80)	–	0.894
EA vs PCG	1	**-2.36(-3.43, -1.29)**	–	0.000

The bold font indicates a statistical difference. EA, Ear Acupressure; APR, Acupressure; MOX, Moxibustion; PCG, Placebo Control Group; ‘8’SRAM, ‘8’Shaped Ring Acupuncture Method; EA+MOX, Ear Acupressure Combined with Moxibustion; HCE, Hypodermic Catgut Embedding; ACU, Acupuncture; AM, Auricular Microneedle; CWMG, Conventional Western Medicine Group; BCG, Blank Control Group.

### 3.4 Network meta-analysis

#### 3.4.1 Network evidence plot

**Figure 3:**
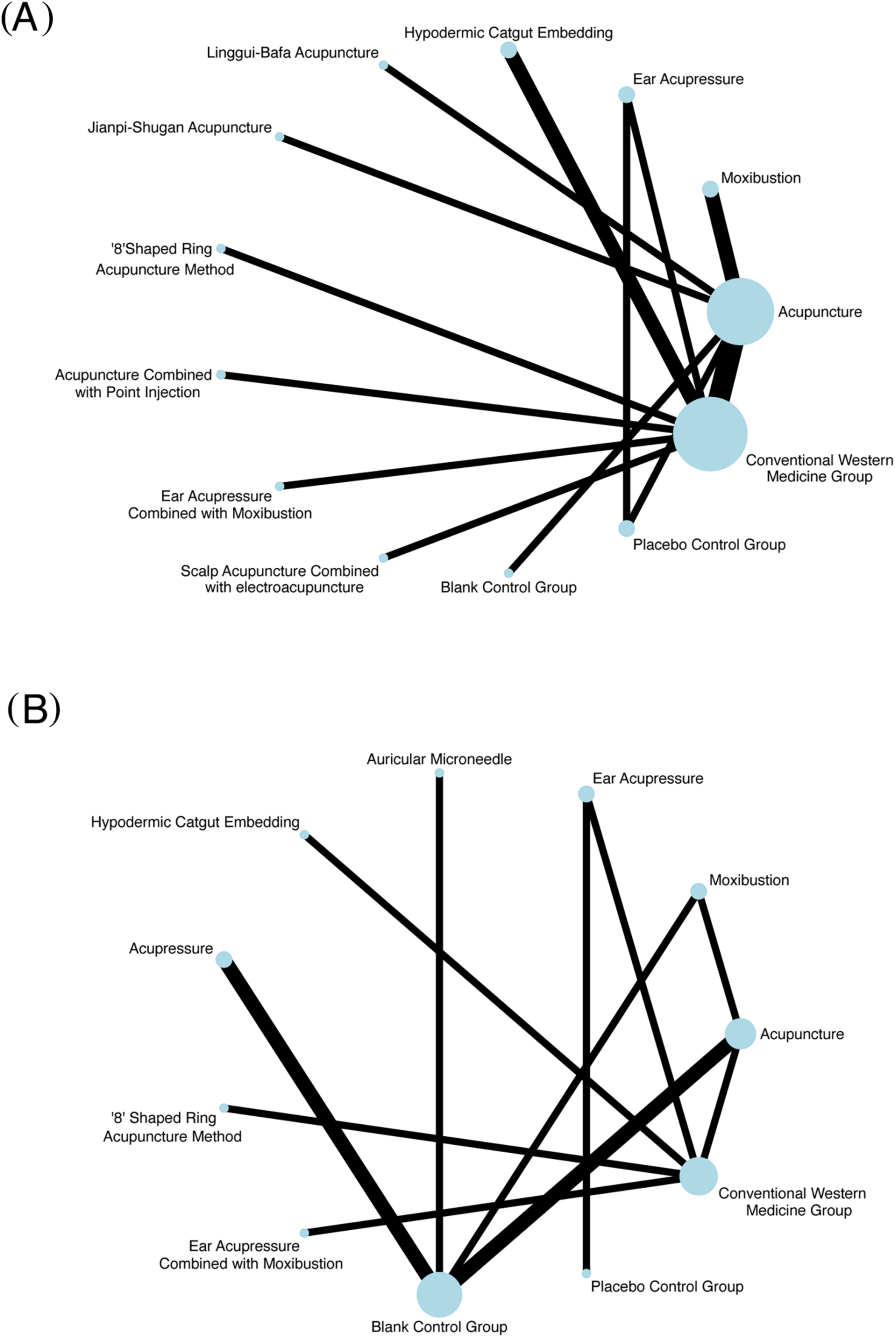
Network evidence plot. **(A)** Overall effective rate. **(B)** Symptom and sign scores.

**Figure 3** shows the network evidence of each outcome indicator. Within the figure: The size of each node is proportional to the sample size of the corresponding intervention. The thickness of each connecting line (edge) is proportional to the number of studies providing direct comparisons between the linked interventions. Consequently, larger nodes and thicker edges indicate interventions with larger sample sizes and direct comparisons supported by a greater number of studies, respectively. The presence of a connecting line between two interventions signifies the availability of direct comparison data. The absence of a connecting line indicates a lack of direct comparative evidence for those interventions. Data on the overall effective rate for PMS improvement using different external TCM therapies were derived from 17 RCTs, encompassing a total of 1,110 patients. These trials evaluated thirteen distinct interventions, including acupuncture, moxibustion, ear acupressure, hypodermic catgut embedding, Linggui-Bafa acupuncture, Jianpi-Shugan acupuncture, ‘8’shaped ring acupuncture method, acupuncture combined with point injection, ear acupressure combined with moxibustion, scalp acupuncture combined with electroacupuncture, blank control group, placebo control group and conventional Western medicine group. **Figure 3A** shows that the sample size of conventional Western medicine group is the largest, and the comparison frequency between acupuncture and conventional Western medicine group is the highest. Symptom and sign scores come from 11 randomized controlled trials with a total of 708 patients, involving 11 intervention measures, including ear acupressure, acupressure, moxibustion, placebo control group, ‘8’shaped ring acupuncture method, ear acupressure combined with moxibustion, hypodermic catgut embedding, acupuncture, auricular microneedle, conventional western medicine group, and blank control group (**Figure 3B**).

We assessed inconsistency within the network using two methods: loop inconsistency analysis to evaluate inconsistency in closed loops, and the node-splitting approach for local inconsistency testing. Both analyses yielded non-significant results (p > 0.05), indicating no significant statistical inconsistency. This demonstrates consistency between the direct and indirect evidence within the network.

#### 3.4.2 Network meta-analysis of overall effective rate

The NMA results demonstrate that acupuncture, moxibustion, ear acupressure, hypodermic catgut embedding, Linggui-Bafa acupuncture, Jianpi-Shugan acupuncture, the ‘8’-shaped ring acupuncture method, acupuncture combined with point injection, ear acupressure combined with moxibustion, and scalp acupuncture combined with electroacupuncture all exhibit significantly greater overall effective rate than both blank control and placebo control groups. Acupuncture, moxibustion, hypodermic catgut embedding, Linggui-Bafa acupuncture, Jianpi-Shugan acupuncture, the ‘8’-shaped ring acupuncture method, acupuncture combined with point injection, and scalp acupuncture combined with electroacupuncture demonstrated significantly higher overall effective rates compared to the conventional Western medication group. Furthermore, Linggui-Bafa acupuncture, Jianpi-Shugan acupuncture, and moxibustion demonstrated significantly superior therapeutic efficacy compared to conventional acupuncture (**Table 3**).

**Table 3:** Overall effective rate: league table.

**JPSG**												
1.20 (0.78,1.86)	**LGBF**											
1.26 (0.84,1.87)	1.05 (0.80,1.37)	**MOX**										
1.42 (0.83,2.42)	1.18 (0.75,1.84)	1.13 (0.75,1.70)	**‘8’SRAM**									
**1.58 (1.09,2.30)**	**1.32 (1.05,1.65)**	**1.26 (1.09,1.45)**	1.12 (0.76,1.64)	**ACU**								
1.64 (1.00,2.69)	1.37 (0.92,2.03)	1.31 (0.91,1.86)	1.16 (0.74,1.81)	1.04 (0.75,1.44)	**SA+ETA**							
1.68 (1.00,2.83)	1.40 (0.91,2.15)	1.34 (0.90,1.98)	1.19 (0.74,1.91)	1.06 (0.74,1.53)	1.02 (0.67,1.57)	**EA**						
**1.87 (1.22,2.86)**	**1.56 (1.14,2.12)**	**1.49 (1.15,1.92)**	1.32 (0.91,1.91)	1.18 (0.96,1.46)	1.14 (0.84,1.55)	1.11 (0.78,1.58)	**HCE**					
**1.88 (1.21,2.94)**	**1.57 (1.12,2.19)**	**1.50 (1.13,1.99)**	1.33 (0.90,1.97)	1.19 (0.93,1.52)	1.15 (0.82,1.60)	1.12 (0.77,1.63)	1.01 (0.81,1.26)	**ACU+PI**				
**2.01 (1.29,3.12)**	**1.67 (1.20,2.32)**	**1.59 (1.20,2.11)**	1.42 (0.96,2.09)	1.27 (1.00,1.61)	1.22 (0.88,1.70)	1.19 (0.82,1.73)	1.07 (0.87,1.33)	1.06 (0.83,1.37)	**EA+MOX**			
**2.33 (1.55,3.50)**	**1.94 (1.46,2.56)**	**1.85 (1.48,2.30)**	**1.64 (1.16,2.33)**	**1.47 (1.24,1.74)**	**1.42 (1.07,1.88)**	1.38 (1.00,1.92)	**1.25 (1.10,1.42)**	**1.23 (1.03,1.48)**	1.16 (0.98,1.38)	**CWMG**		
**3.85 (2.02,7.32)**	**3.20 (1.81,5.68)**	**3.06 (1.77,5.27)**	**2.72 (1.47,5.01)**	**2.43 (1.44,4.11)**	**2.34 (1.32,4.17)**	**2.29 (1.54,3.39)**	**2.06 (1.23,3.46)**	**2.04 (1.20,3.48)**	**1.92 (1.13,3.26)**	1.65 (1.00,2.73)	**PCG**	
**96.35 (6.01,1545.08)**	**80.21 (5.08,1266.23)**	**76.59 (4.88,1202.34)**	**68.03 (4.23,1093.14)**	**60.85 (3.89,951.74)**	**58.69 (3.68,935.70)**	**57.32 (3.58,918.28)**	**51.56 (3.27,812.87)**	**51.12 (3.23,808.38)**	**48.04 (3.04,759.24)**	**41.41 (2.63,650.95)**	**25.04 (1.52,411.68)**	**BCG**

The bold font indicates a statistical difference. JPSG, Jianpi-Shugan Acupuncture; LGBF, Linggui-Bafa Acupuncture; SA+ETA, Scalp Acupuncture Combined with electroacupuncture; ACU+PI, Acupuncture Combined with Point Injection; EA, Ear Acupressure; MOX, Moxibustion; PCG, Placebo Control Group; ‘8’SRAM, ‘8’shaped Ring Acupuncture Method; EA+MOX, Ear Acupressure Combined with Moxibustion; HCE, Hypodermic Catgut Embedding; ACU, Acupuncture; CWMG, Conventional Western Medicine Group; BCG, Blank Control Group.

**Figure 4:**
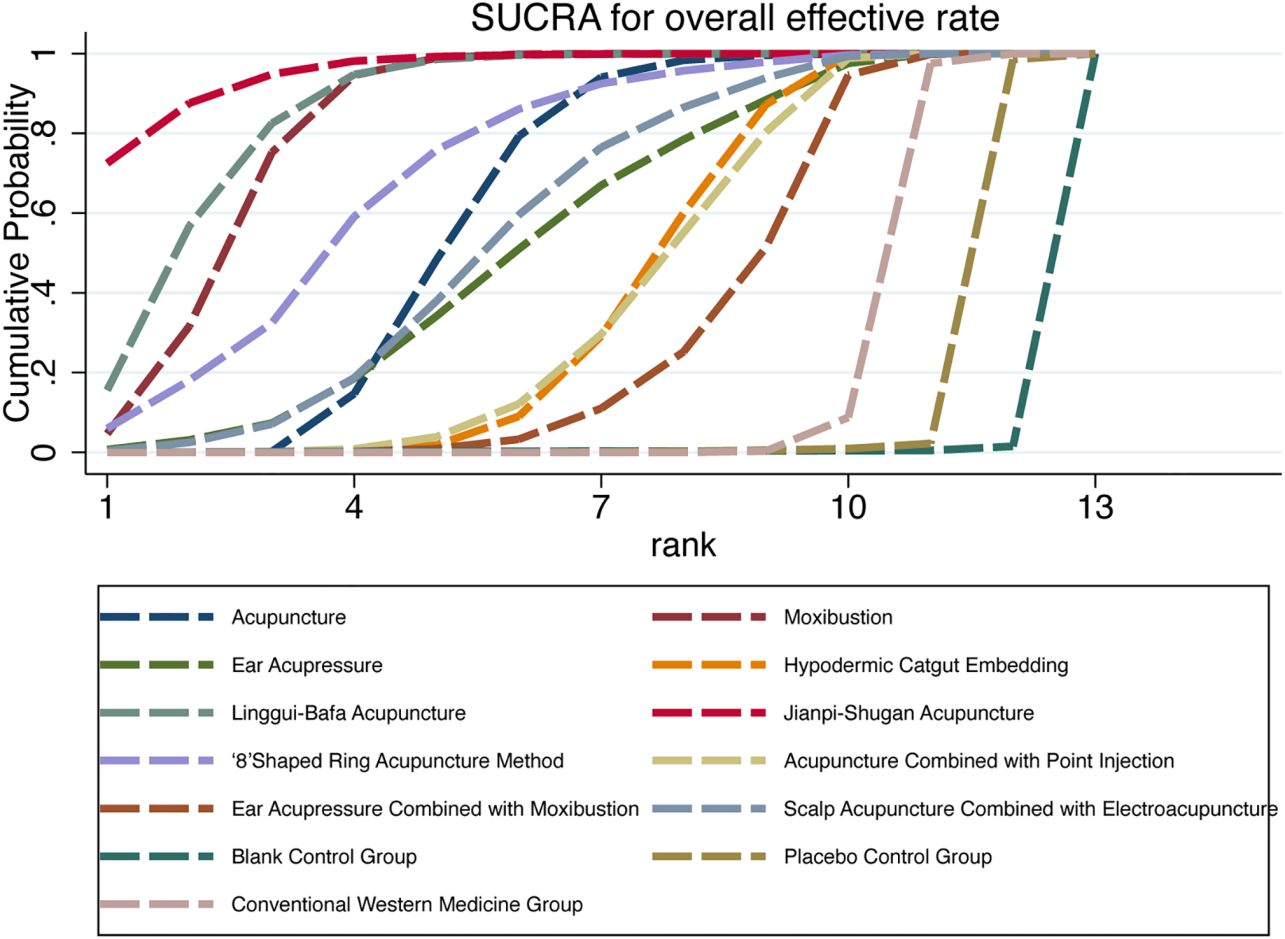
Cumulative probability plot of overall effective rate.

Higher SUCRA values indicate superior intervention efficacy and ranking position. Based on our findings, Jianpi-Shugan acupuncture (SUCRA = 95.7%) demonstrated the highest probability of being the optimal intervention for improving overall PMS treatment response rates, while the blank control group (SUCRA = 0.3%) showed the lowest probability. The mean SUCRA values and corresponding rankings for all thirteen interventions are presented in **Table 4** and **Figure 4**.

**Table 4:** SUCRA values of overall effective rate and ranking of interventions.

Rank	SUCRA	Treatments
1	95.7	Jianpi-Shugan acupuncture
2	87.3	Linggui-Bafa acupuncture
3	84.1	Moxibustion
4	72	‘8’shaped ring acupuncture method
5	61.4	Acupuncture
6	57	Scalp Acupuncture Combined with electroacupuncture
7	53.9	Ear Acupressure
8	40.6	Hypodermic Catgut Embedding
9	39.8	Acupuncture Combined with Point Injection
10	32.3	Ear Acupressure Combined with moxibition
11	17.2	Conventional Western Medicine Group
12	8.5	Placebo Control Group
13	0.3	Blank Control Group

#### 3.4.3 Network meta-analysis of symptom and sign scores

The NMA of symptom severity scores demonstrated that acupressure significantly outperformed the blank control group in reducing symptom scores. No other comparisons reached statistical significance (**Table 5**).

**Table 5:** Symptom and sign scores: league table.

**EA**										
-0.35 (-23.84,23.14)	**APR**									
-2.38 (-25.06,20.30)	-2.03 (-18.99,14.93)	**MOX**								
-2.37 (-14.88,10.14)	-2.02 (-28.60,24.56)	0.01 (-25.87,25.88)	**PCG**							
-3.56 (-22.09,14.96)	-3.21 (-26.38,19.96)	-1.18 (-23.52,21.16)	-1.19 (-23.53,21.15)	‘8’**SRAM**						
-3.66 (-22.06,14.74)	-3.31 (-26.39,19.76)	-1.28 (-23.52,20.96)	-1.29 (-23.53,20.95)	-0.10 (-18.03,17.83)	**EA+MOX**					
-5.40 (-23.77,12.98)	-5.05 (-28.10,18.00)	-3.02 (-25.24,19.20)	-3.03 (-25.24,19.19)	-1.84 (-19.74,16.07)	-1.74 (-19.52,16.04)	**HCE**				
-5.77 (-24.03,12.49)	-5.42 (-20.27,9.43)	-3.39 (-16.88,10.09)	-3.40 (-25.50,18.70)	-2.21 (-20.03,15.62)	-2.11 (-19.81,15.59)	-0.37 (-18.05,17.30)	**ACU**			
-7.29 (-31.48,16.91)	-6.94 (-23.39,9.51)	-4.91 (-22.99,13.18)	-4.91 (-32.13,22.30)	-3.72 (-27.61,20.16)	-3.62 (-27.41,20.16)	-1.89 (-25.66,21.88)	-1.52 (-17.43,14.40)	**AM**		
-10.66 (-24.08,2.76)	-10.31 (-29.66,9.05)	-8.28 (-26.63,10.07)	-8.29 (-26.61,10.04)	-7.10 (-19.86,5.67)	-7.00 (-19.59,5.59)	-5.26 (-17.81,7.29)	-4.89 (-17.35,7.58)	-3.37 (-23.57,16.83)	**CWMG**	
-14.39 (-35.13,6.35)	**-14.04 (-24.74, -3.34)**	-12.01 (-25.09,1.08)	-12.02 (-36.21,12.17)	-10.83 (-31.19,9.54)	-10.73 (-30.98,9.53)	-8.99 (-29.22,11.24)	-8.62 (-18.50,1.27)	-7.10 (-19.59,5.38)	-3.73 (-19.62,12.16)	**BCG**

The bold font indicates a statistical difference. EA, Ear Acupressure; APR, Acupressure; MOX, Moxibustion; PCG, Placebo Control Group; ‘8’SRAM, ‘8’shaped ring acupuncture method; EA+MOX, Ear Acupressure Combined with Moxibustion; HCE, Hypodermic Catgut Embedding; ACU, Acupuncture; AM, Auricular Microneedle; CWMG, Conventional Western; BCG, Blank Control Group.

**Figure 5:**
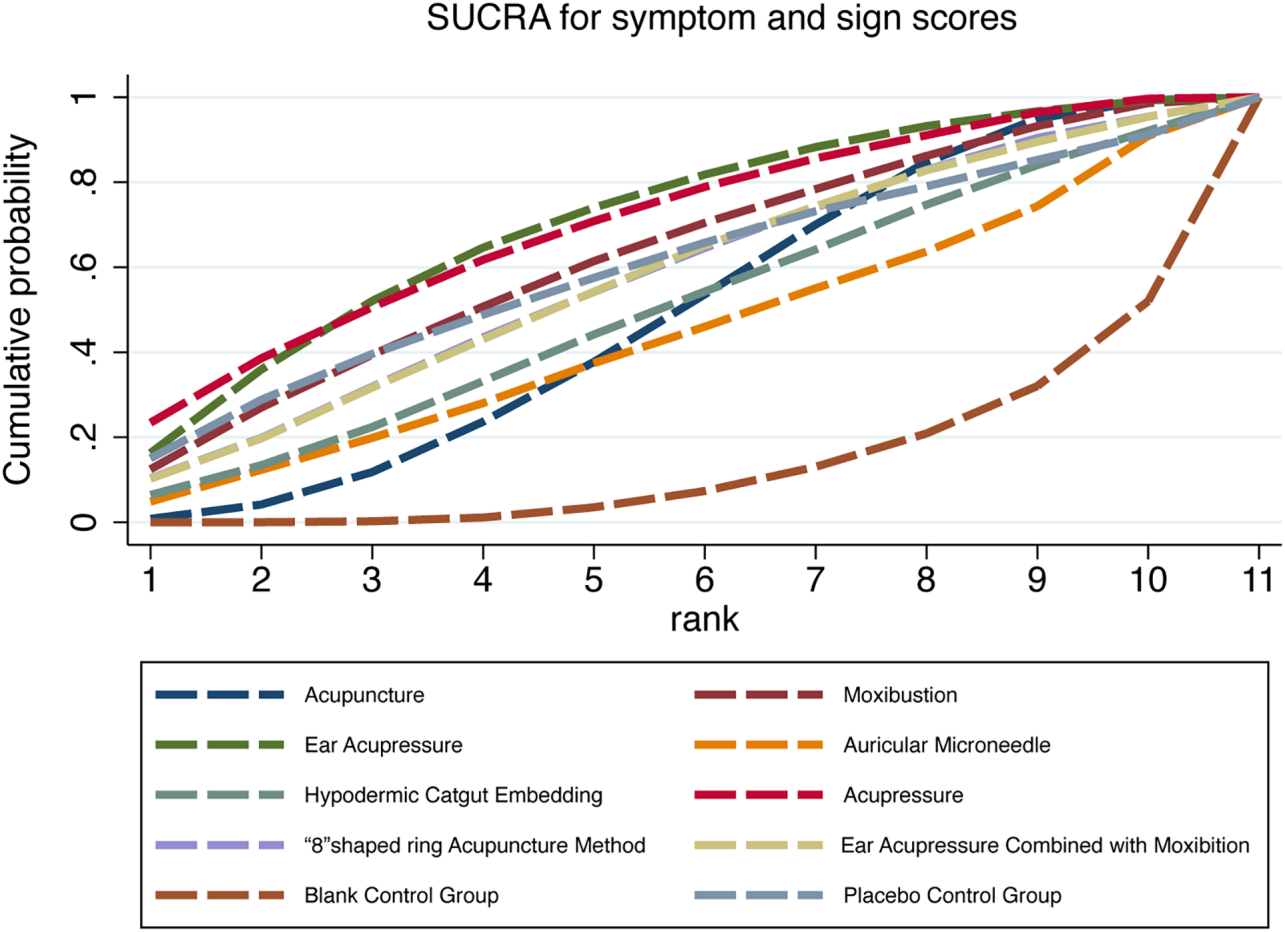
Cumulative probability plot of symptom and sign scores.

As presented in **Table 6** and **Figure 5**, ear acupressure achieved the highest SUCRA value among all interventions. The efficacy ranking hierarchy was as follows: ear acupressure, acupressure, moxibustion, placebo control group, ‘8’shaped ring acupuncture method, ear acupressure combined with moxibustion, hypodermic catgut embedding, acupuncture, auricular microneedle, conventional Western medicine group, and blank control group. These results indicate that ear acupressure may represent the optimal intervention for reducing symptom and sign scores in PMS treatment.

**Table 6:** SUCRA values of symptom and sign scores and ranking of interventions.

Rank	SUCRA	Treatments
1	71.2	Ear Acupressure
2	69.4	Acupressure
3	61.7	Moxibustion
4	59.4	Placebo Control Group
5	57	“8”shaped ring acupuncture method
6	56.4	Ear Acupressure Combined with moxibustion
7	48.6	Hypodermic Catgut Embedding
8	47.5	Acupuncture
9	42.8	Auricular Microneedle
10	23.2	Conventional Western Medicine Group
11	12.8	Blank Control Group

### 3.5 Adverse events

Among the 21 included studies, two reported adverse events. Habek documented a single adverse reaction—local subcutaneous hematoma—in the intervention group at 5.56% incidence. Fan reported two exclusive control-group adverse reactions: gastrointestinal disturbances and migraine, occurring at 45.83% incidence. Our analysis identified acupuncture-associated localized subcutaneous hematoma and conventional Western medication-related gastrointestinal disturbances/migraine as predominant adverse reactions. No serious adverse events emerged in the evidence base.

### 3.6 Publication bias

**Figure 6:**
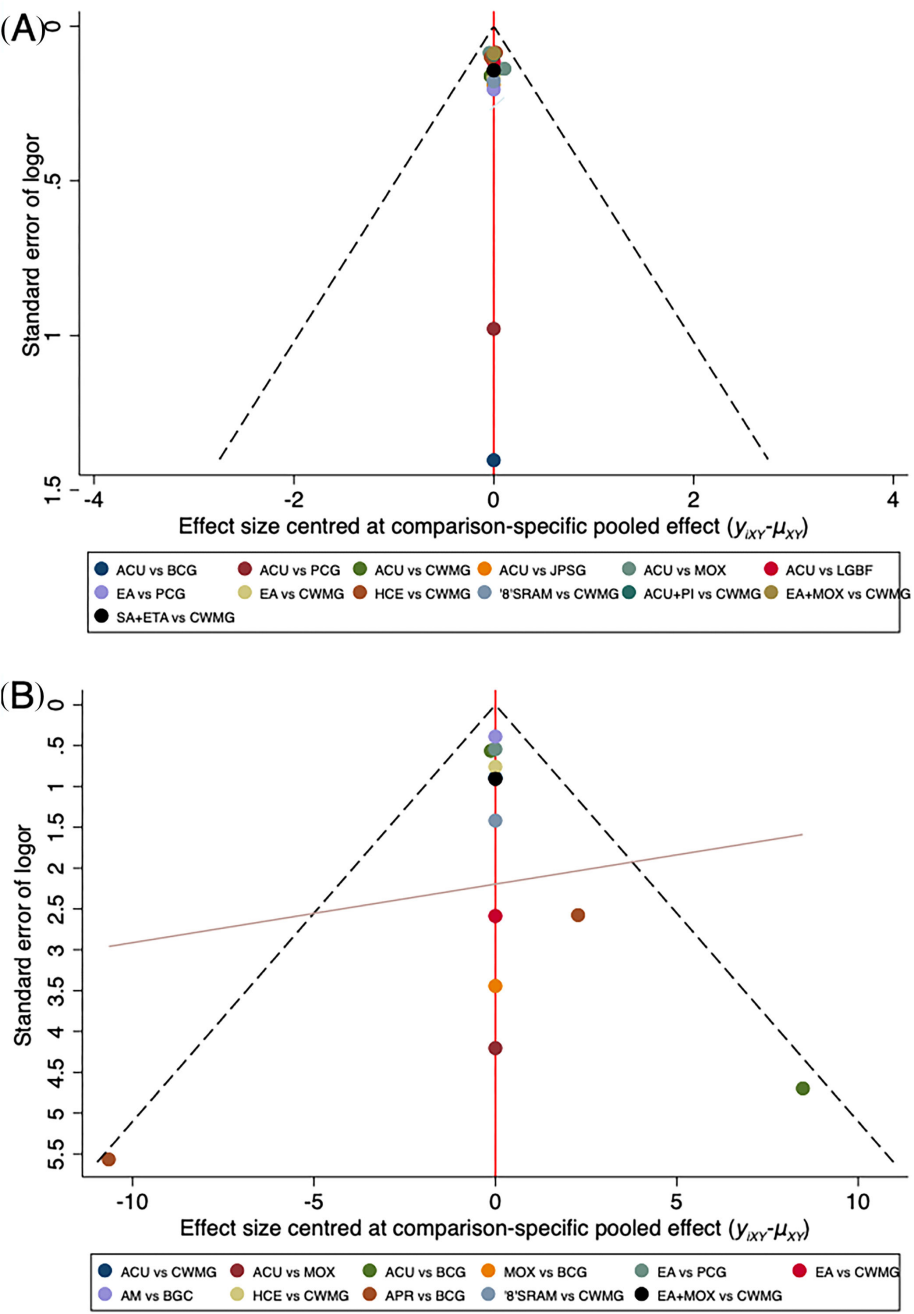
Funnel plot of overall effective rate **(A)** and symptom and sign scores **(B)**. EA, Ear Acupressure; APR, Acupressure; MOX, Moxibustion; PCG, Placebo Control Group; '8'SRAM, '8'Shaped Ring Acupuncture Method; EA+MOX, Ear Acupressure Combined with Moxibustion; HCE, Hypodermic Catgut Embedding; ACU, Acupuncture; SA+ETA, Scalp Acupuncture Combined with Electroacupuncture; JPSG, Jianpi-Shugan Acupuncture; LGBF, Linggui-Bafa Acupuncture; ACU+PI, Acupuncture Combined with Point Injection; AM, Auricular Microneedle; CWMG, Conventional Western Medicine Group; BCG, Blank Control Group.

Assessment of publication bias for both the overall effective rate and symptom severity scores revealed low likelihood of bias, as data points clustered within the funnel plot triangle and distributed symmetrically across the null line (**Figures 6A, B**). However, formal Begg’s tests yielded differential results: while no significant publication bias was detected for symptom severity scores (p > 0.05), the overall effective rate analysis suggested potential susceptibility to publication bias or small-study effects (**Supplementary Figures S2, S3**).

## 4 Discussion

PMS is a menstrual cycle-related disorder characterized by a constellation of recurrent cyclic physical, emotional, and behavioral symptoms. These symptoms emerge during the late luteal phase of the menstrual cycle and typically subside soon after the onset of menstruation (17). Our findings indicate that conventional Western medication represents the most prevalent control intervention, confirming real-world practice patterns. While SSRIs and ovulation suppression constitute first-line pharmacotherapies for premenstrual syndrome, their therapeutic utility remains suboptimal due to clinically significant adverse effects that may compromise patient safety (18). This clinical demand has driven interest in complementary management strategies that offer durable efficacy alongside a benign side-effect profile. External TCM therapies represent a promising yet systematically underexplored category within this domain. While prior meta-analyses have confirmed acupuncture’s efficacy in PMS (13, 14), a comprehensive comparative evaluation of diverse TCM external modalities is lacking. This study therefore aims to fill this gap through a network meta-analysis. Given the diverse modalities within external TCM therapies, determining the optimal intervention or combination became our primary research objective. Through rigorous implementation of our predefined study protocol—including comprehensive literature searches and systematic screening of publications on external TCM therapies for PMS—we ultimately identified 21 RCTs meeting inclusion criteria. All evaluated acupuncture interventions in the included trials were performed by licensed or appropriately trained practitioners, ensuring the clinical validity and safety context of our findings. We subsequently categorized the interventional approaches employed across these 21 RCTs as follows: acupuncture, moxibustion, ear acupressure, acupressure, ‘8’shaped ring acupuncture method, ear acupressure combined with moxibustion, hypodermic catgut embedding, auricular microneedle, scalp acupuncture combined with electroacupuncture, Jianpi-Shugan acupuncture, Linggui-Bafa acupuncture, acupuncture combined with point injection. We analyzed two outcome measures, total effective rate and symptom and sign scores, providing direct and indirect evidence for the optimal treatment plan related to PMS using TCM external treatment methods. The direct evidence of overall effective rate indicates that, Jianpi-Shugan acupuncture, Linggui-Bafa acupuncture, and moxibustion are superior to acupuncture; acupuncture, ‘8’shaped ring acupuncture method, scalp acupuncture combined with electroacupuncture, ear acupressure, hypodermic catgut embedding, and acupuncture combined with point injection are better than conventional Western medicine group. For symptom and sign scores, acupuncture, ‘8’shaped ring acupuncture method, ear acupressure, hypodermic catgut embedding, and ear acupressure combined with moxibustion demonstrated significantly greater improvement than conventional Western medicine group; acupuncture, moxibustion, acupressure, and auricular microneedle demonstrated significantly superior efficacy compared to the blank control group. However, significant heterogeneity was observed in some outcomes (I² > 50%). Upon examining the original RCTs, we determined this heterogeneity likely stems from clinical design variations, including differences in: acupoint selection; needling depth; treatment duration; control group medication protocols. Our NMA ranking probabilities for overall PMS effective rates indicated Jianpi-Shugan acupuncture had the highest probability of being optimal (SUCRA = 95.7%). For symptom severity scores, ear acupressure demonstrated superior reduction efficacy compared to other interventions. Nevertheless, after synthesizing direct and indirect evidence, most comparative analyses between interventions showed non-significant differences. These findings require cautious interpretation due to substantial heterogeneity. We conducted exploratory analyses regarding TCM external therapies’ influence on emotional manifestations of PMS—particularly depression and anxiety. Regrettably, inconsistent measurement units across RCTs precluded quantitative synthesis. Consequently, emotional domain improvements were not analyzed in this study. Although the adverse events caused by acupuncture and moxibustion are relatively minor (19), Several adverse events were reported across studies, with three types demonstrating notable incidence: gastrointestinal disturbances, migraine, and localized subcutaneous hematoma. The former two events were likely attributable to conventional Western medication side effects. Regarding hematoma occurrence in needle-averse individuals, implementing pre-acupuncture counseling to explain expected physiological responses and meticulous avoidance of superficial veins during needle insertion may alleviate patient anxiety and reduce incidence rates.

Research in animal models reveals an association between menstrual cycle-associated hippocampal dysfunction and the pathogenesis of PMS (20, 21). Reliable detection of hippocampal abnormalities during the late luteal phase in women with premenstrual syndrome is critical for understanding underlying neural mechanisms. Moreover, the amygdala serves as a key neural substrate within extensive networks regulating emotional and pain processing. Dysfunction in this region contributes significantly to emotional and physical disorders (22). Aberrant amygdala functional connectivity is implicated in PMS (23). The amygdala likely plays a key role in PMS development, progression, and symptom relief. Multiple fMRI studies show acupuncture triggers broader neural responses in amygdala-related brain networks (24, 25). Seed-based resting-state functional connectivity (FC) analysis is a well-established functional brain imaging method for investigating temporal dependencies between a target brain region and other areas throughout the brain (26, 27). Qin et al. have confirmed FC analysis as a sensitive method for detecting acupuncture’s effects on amygdala-related brain networks. Their findings reveal significant and specific fMRI signal changes in the amygdala during acupuncture stimulation (25). Electroacupuncture stimulation at SP6 may downregulate abnormal amygdala functional connectivity in PMS patients, likely attributable to SP6’s therapeutic properties. Acupuncture significantly alleviates core PMS symptoms, including both physical and emotional manifestations (22). Previous research indicates acupuncture primarily modulates patients’ physiological responses through the central nervous system (28). More specifically, acupuncture may alleviate PMS symptoms by normalizing aberrant neural responses within this system. Furthermore, growing evidence links PMS pathophysiology with inflammatory processes (29). Although this research area remains evolving, substantial evidence has established connections between PMS and psychological stress—a factor closely associated with inflammatory responses. Puder et al. assessed relationships between inflammatory markers and physical/psychological symptoms across the menstrual cycle in normal-weight and overweight women. Their findings demonstrated that fluctuations in serum TNF-α and hs-CRP levels were associated with these symptoms throughout the menstrual cycle (30). Another study in young women demonstrated that PMS patients had significantly higher levels of interleukin cytokines including IL-4, IL-10, IL-12, and IFN-γ compared to healthy controls (31). We therefore hypothesize that stress-induced inflammation constitutes a core pathogenic mechanism in PMS (32). Current evidence suggests this inflammatory response involves integrated peripheral and central pathways: Stress activates the peripheral immune system to release pro-inflammatory cytokines, which compromise the blood-brain barrier. This breach enables peripheral immune cell infiltration into the central nervous system, ultimately triggering neuroinflammation (33). Collectively, these findings establish a theoretical foundation for our results and offer valuable reference frameworks for subsequent research.

Our study has several important limitations. First, certain comparisons relied on limited evidence, with some interventions supported by only a single study. These findings therefore require cautious interpretation. Second, our comprehensive inclusion strategy incorporated broad study variations, inevitably introducing clinical heterogeneity, notably in treatment protocols. Future studies should standardize methodologies, particularly concerning measurement tools and participant selection criteria, to minimize heterogeneity. Third, few studies stratified PMS severity using standardized classifications such as mild, moderate or severe. Most merely confirmed PMS diagnosis without symptom severity grading, limiting our analysis to overall treatment effects. Fourth, we observed substantial variability in control group medications and inconsistent application of TCM techniques regarding point selection, needling depth and duration. This methodological heterogeneity directly impacted outcome consistency. Fifth, most studies lacked follow-up assessments, precluding evaluation of long-term efficacy. Regarding safety, only two studies descriptively reported adverse events, making quantitative safety analysis unfeasible. Finally, inherent NMA limitations include statistical complexity and inconsistency management, challenges that require advanced methodological approaches.

In summary, this systematic review and NMA consolidates current evidence on TCM external therapies, offering conceptual foundations for clinical implementation and scholarly investigation.

## 5 Conclusion

Our NMA suggests that TCM external therapies for PMS present a promising tolerability, with reported adverse events being predominantly mild. Jianpi-Shugan acupuncture was identified as the optimal choice for overall treatment response, and Ear Acupressure was most effective in alleviating quantitative symptom burdens. These comparative rankings, however, are tempered by the sparsity of direct evidence and methodological constraints of the included trials; thus, they do not support definitive conclusions. Conclusive evidence will require head-to-head trials specifically powered to compare these interventions.

## Data Availability

The original contributions presented in the study are included in the article/**Supplementary Material**. Further inquiries can be directed to the corresponding authors.
