# Supplementary material for: Efficacy comparison of different external traditional Chinese medicine therapies as monotherapy or in combination for premenstrual syndrome: a systematic review and network meta-analysis
Source: Front Psychiatry. 2026 Feb 4;17:1720232. doi: 10.3389/fpsyt.2026.1720232 (PMC12913161; doi:10.3389/fpsyt.2026.1720232)
Supplement: Supplementary file 1 [file Table1.docx]

**SUPPLEMENTARY MATERIAL**

**Table of Contents**

**Supplementary Methods 1 –** Database search strategy.

**Supplementary Methods** **2 –** Conversion formula for standard deviation (SD).

**Supplementary Methods 3** **–** Explanation of technical terms.

**Supplementary Methods 4 –** Inclusion in the list of studies.

**Supplementary Table S1** **–** Study characteristics of the included studies.

**Supplementary Figure S1 –** Excluded studies.

**Supplementary Table S2** **–** Risk of bias table of included studies.

**Supplementary Figure S2–** The results of the Begg test for overall effective rate.

**Supplementary Figure S3–** The results of the Begg test for symptom and sign scores.

**Supplementary Table S3 –** List of Abbreviations.

**Supplementary Methods 1 –** Database search strategy.

**PubMed**

**Date Run: 17/03/2025 22:58:55**

| #1 | (((((((((((((((((((((((((((((((((electroacupuncture) OR (acupuncture)) OR (acupuncture therapy)) OR (auricular acupuncture)) OR (auricular needle)) OR (ear acupuncture)) OR (auricular plaster therapy)) OR (transcutaneous electric nerve stimulation)) OR (electric stimulation therapy)) OR (laser acupuncture)) OR (auricular point sticking)) OR (acupressure)) OR (dry needle)) OR (scalp acupuncture)) OR (scalp sensory)) OR (scalp stimulation)) OR (filliform needle)) OR (moxibustion)) OR (moxabustion)) OR (filiform needle)) OR (acupuncture injection)) OR (pharmacopuncture)) OR (pharmacoacupuncture))) OR (catgut embedding)) OR (acupoint catgut embedding therapy)) OR (catgut implantation)) OR (catgut embedding)) OR (acupoint injection)) OR (point injection)) OR (body acupuncture)) OR (acupoint injection)) OR (point injection)) OR (abdominal acupuncture)) OR (navel acupuncture) | 206,047 |
| --- | --- | --- |
| #2 | ((((((premenstrual syndrome) OR (menstrual distress)) OR (menstrual symptoms)) OR (premenstrual stress)) OR (premenstrual tension)) OR (premenstrual tension syndrome)) OR (premenstruation syndrome) | 39,514 |
| #3 | (((((((premenstrual syndrome) OR (menstrual distress)) OR (menstrual symptoms)) OR (premenstrual stress)) OR (premenstrual tension)) OR (premenstrual tension syndrome)) OR (premenstruation syndrome)) AND ((((((((((((((((((((((((((((((((((electroacupuncture) OR (acupuncture)) OR (acupuncture therapy)) OR (auricular acupuncture)) OR (auricular needle)) OR (ear acupuncture)) OR (auricular plaster therapy)) OR (transcutaneous electric nerve stimulation)) OR (electric stimulation therapy)) OR (laser acupuncture)) OR (auricular point sticking)) OR (acupressure)) OR (dry needle)) OR (scalp acupuncture)) OR (scalp sensory)) OR (scalp stimulation)) OR (filliform needle)) OR (moxibustion)) OR (moxabustion)) OR (filiform needle)) OR (acupuncture injection)) OR (pharmacopuncture)) OR (pharmacoacupuncture))) OR (catgut embedding)) OR (acupoint catgut embedding therapy)) OR (catgut implantation)) OR (catgut embedding)) OR (acupoint injection)) OR (point injection)) OR (body acupuncture)) OR (acupoint injection)) OR (point injection)) OR (abdominal acupuncture)) OR (navel acupuncture)) | 460 |

**Cochrane**

| **Date Run:** | | **18/03/2025 14:49:55** |  | |
| --- | --- | --- | --- | --- |
| #1 | MeSH descriptor: [Electroacupuncture] explode all trees | | | 1170 |
| #2 | MeSH descriptor: [Acupuncture] explode all trees | | | 216 |
| #3 | (Pharmacopuncture):ti,ab,kw OR (acupuncture therapy):ti,ab,kw OR (acupuncture):ti,ab,kw OR (herb acupuncture):ti,ab,kw OR (herbal acupuncture):ti,ab,kw | | | 21149 |
| #4 | MeSH descriptor: [Acupuncture, Ear] explode all trees | | | 267 |
| #5 | (Ear Acupuncture):ti,ab,kw OR (auricular acupuncture):ti,ab,kw OR (auricular needle):ti,ab,kw OR (auricular plaster therapy):ti,ab,kw | | | 1565 |
| #6 | MeSH descriptor: [Transcutaneous Electric Nerve Stimulation] explode all trees | | | 2858 |
| #7 | (Electroanalgesia):ti,ab,kw OR (Percutaneous Electrical Neuromodulation):ti,ab,kw OR (Percutaneous Neuromodulation Therapy):ti,ab,kw OR (Analgesic Cutaneous Electrostimulation):ti,ab,kw OR (Transcutaneous Nerve Stimulation):ti,ab,kw | | | 5080 |
| #8 | (Percutaneous Electric Nerve Stimulation):ti,ab,kw OR (Transcutaneous Electric Stimulation):ti,ab,kw OR (TENS):ti,ab,kw OR (Percutaneous Electrical Nerve Stimulation):ti,ab,kw OR (Transcutaneous Electrical Stimulation):ti,ab,kw | | | 6270 |
| #9 | (Transcutaneous Electrical Nerve Stimulation):ti,ab,kw OR (Transdermal Electrostimulation):ti,ab,kw OR (electric stimulation therapy):ti,ab,kw | | | 7736 |
| #10 | (laser acupuncture):ti,ab,kw OR (auricular point sticking):ti,ab,kw OR (acupressure):ti,ab,kw | | | 3309 |
| #11 | MeSH descriptor: [Dry Needling] explode all trees | | | 204 |
| #12 | (scalp acupuncture):ti,ab,kw OR (scalp sensory):ti,ab,kw OR (scalp stimulation):ti,ab,kw OR (filliform needle):ti,ab,kw | | | 1524 |
| #13 | MeSH descriptor: [Moxibustion] explode all trees | | | 681 |
| #14 | (moxabustion):ti,ab,kw OR (moxibustion):ti,ab,kw OR (point injection):ti,ab,kw OR (abdominal acupuncture):ti,ab,kw OR (navel acupuncture):ti,ab,kw | | | 20578 |
| #15 | (acupuncture injection):ti,ab,kw OR (acupoint injection):ti,ab,kw OR (point injection):ti,ab,kw OR (body acupuncture):ti,ab,kw OR (acupoint injection):ti,ab,kw | | | 19858 |
| #16 | MeSH descriptor: [Acupuncture] explode all trees | | | 216 |
| #17 | (pharmacopuncture):ti,ab,kw OR (pharmacoacupuncture):ti,ab,kw OR (catgut embedding):ti,ab,kw OR (acupoint catgut embedding therapy):ti,ab,kw OR (catgut implantation):ti,ab,kw | | | 449 |
| #18 | MeSH descriptor: [Premenstrual Syndrome] explode all trees | | | 678 |
| #19 | (premenstrual syndrome):ti,ab,kw OR (menstrual distress):ti,ab,kw OR (menstrual symptoms):ti,ab,kw OR (premenstrual stress):ti,ab,kw OR (premenstrual tension):ti,ab,kw | | | 3632 |
| #20 | (premenstrual tension syndrome):ti,ab,kw OR (premenstruation syndrome):ti,ab,kw | | | 237 |
| **#21** | **#1 OR #2 OR #3 OR #4 OR #5 OR #6 OR #7 OR #8 OR #9 OR #10 OR #11 OR #12 OR #13 OR #14 OR #15 OR #16 OR #17** | | | **52134** |
| **#22** | **#18 OR #19 OR #20** | | | **3647** |
| **#23** | **#21 AND #22** | | | **267** |

**Embase**

**Date Run: March 18, 2025**

| #1 | 'electroacupuncture':ab,ti OR 'electric acupuncture':ab,ti OR 'electrical acupoint stimulation':ab,ti OR 'electrical acupuncture':ab,ti OR 'electrode acupuncture':ab,ti OR 'electronic acupuncture':ab,ti | 9953 |
| --- | --- | --- |
| #2 | 'acupuncture':ab,ti OR 'acupuncture therapy':ab,ti OR 'shonishin':ab,ti | 41950 |
| #3 | 'auricular acupuncture':ab,ti OR 'auriculoacupuncture':ab,ti OR 'ear acupuncture':ab,ti OR 'earlobe acupuncture':ab,ti OR 'auricular needle':ab,ti OR 'auricular plaster therapy':ab,ti | 1199 |
| #4 | 'transcutaneous electric nerve stimulation':ab,ti OR 'electric stimulation therapy':ab,ti OR 'laser acupuncture':ab,ti OR 'auricular point sticking':ab,ti OR 'acupressure':ab,ti OR 'dry needle':ab,ti OR 'scalp acupuncture':ab,ti OR 'scalp sensory':ab,ti OR 'scalp stimulation':ab,ti OR 'filliform needle':ab,ti OR 'moxibustion':ab,ti OR 'moxabustion':ab,ti OR 'filiform needle':ab,ti OR 'acupuncture injection':ab,ti OR 'pharmacopuncture':ab,ti OR 'pharmacoacupuncture':ab,ti OR 'catgut embedding':ab,ti OR 'acupoint catgut embedding therapy':ab,ti OR 'catgut implantation':ab,ti OR 'acupoint injection':ab,ti OR 'point injection':ab,ti OR 'body acupuncture':ab,ti OR 'abdominal acupuncture':ab,ti OR 'navel acupuncture':ab,ti | 11012 |
| **#5** | **#1 OR #2 OR #3 OR #4** | **52038** |
| #6 | 'premenstrual syndrome':ab,ti OR 'menstrual distress':ab,ti OR 'menstrual symptoms':ab,ti OR 'premenstrual stress':ab,ti OR 'premenstrual tension':ab,ti OR 'premenstrual tension syndrome':ab,ti OR 'premenstruation syndrome':ab,ti | 5083 |
| **#7** | **#5 AND #6** | **81** |

**Web of science**

**Date Run: Tue Mar 18 2025 14:22:35 GMT+0800**

| #36 | ((((((((((((((((((((((((((((((((((((((TS=(auricular acupuncture)) OR TS=(auriculoacupuncture)) OR TS=(ear acupuncture)) OR TS=(earlobe acupuncture)) OR TS=(auricular needle)) OR TS=(auricular plaster therapy)) OR TS=(acupuncture)) OR TS=(acupuncture therapy)) OR TS=(shonishin)) OR TS=(electroacupuncture)) OR TS=(electric acupuncture)) OR TS=(electrical acupoint stimulation)) OR TS=(electrical acupuncture)) OR TS=(electrode acupuncture)) OR TS=(electronic acupuncture)) OR TS=(transcutaneous electric nerve stimulation)) OR TS=(electric stimulation therapy)) OR TS=(laser acupuncture)) OR TS=(auricular point sticking)) OR TS=(acupressure)) OR TS=(dry needle)) OR TS=(scalp acupuncture)) OR TS=(scalp sensory)) OR TS=(scalp stimulation)) OR TS=(filliform needle)) OR TS=(moxibustion)) OR TS=(moxabustion)) OR TS=(filiform needle)) OR TS=(acupuncture injection)) OR TS=(pharmacopuncture)) OR TS=(pharmacoacupuncture)) OR TS=(catgut embedding)) OR TS=(acupoint catgut embedding therapy)) OR TS=(catgut implantation)) OR TS=(acupoint injection)) OR TS=(point injection)) OR TS=(body acupuncture)) OR TS=(abdominal acupuncture)) OR TS=(navel acupuncture) and Preprint Citation Index (Exclude – Database) | 371305 | 371305 |
| --- | --- | --- | --- |
| #37 | ((((((TS=(premenstrual syndrom)) OR TS=(menstrual distress)) OR TS=(menstrual symptoms)) OR TS=(premenstrual stress)) OR TS=(premenstrual tension)) OR TS=(premenstrual tension syndrome)) OR TS=(premenstruation syndrome) and Preprint Citation Index (Exclude – Database) | 18825 | 18825 |
| **#38** | **#37 AND #36 and Preprint Citation Index (Exclude – Database)** | **375** | 375 |

**Chinese National Knowledge Infrastructure (CNKI)**

**Date Run: March 18, 2025**

**Search Details ：**

((SU=(针刺 + 电针 + 针灸 + 激光针 + 体针 + 温针灸 + 毫针 + 干针 + 火针 + 激光穴位照射 + 穴位贴敷 + 针刺治疗 + 针灸疗法 + 激光穴位 + 穴位注射 + 艾灸 + 灸法 + 灸疗 + 灸术 + 腹针 + 脐针 + 穴位埋线 + 耳针 + 耳针疗法 + 耳穴疗法 + 头皮针 + 头针疗法 + 头针 + 穴位按压 + 埋针 + 皮内针 + 经皮穴位电刺激) OR TI=(针刺 + 电针 + 针灸 + 激光针 + 体针 + 温针灸 + 毫针 + 干针 + 火针 + 激光穴位照射 + 穴位贴敷 + 针刺治疗 + 针灸疗法 + 激光穴位 + 穴位注射 + 艾灸 + 灸法 + 灸疗 + 灸术 + 腹针 + 脐针 + 穴位埋线 + 耳针 + 耳针疗法 + 耳穴疗法 + 头皮针 + 头针疗法 + 头针 + 穴位按压 + 埋针 + 皮内针 + 经皮穴位电刺激) OR KY=(针刺 + 电针 + 针灸 + 激光针 + 体针 + 温针灸 + 毫针 + 干针 + 火针 + 激光穴位照射 + 穴位贴敷 + 针刺治疗 + 针灸疗法 + 激光穴位 + 穴位注射 + 艾灸 + 灸法 + 灸疗 + 灸术 + 腹针 + 脐针 + 穴位埋线 + 耳针 + 耳针疗法 + 耳穴疗法 + 头皮针 + 头针疗法 + 头针 + 穴位按压 + 埋针 + 皮内针 + 经皮穴位电刺激) OR AB=(针刺 + 电针 + 针灸 + 激光针 + 体针 + 温针灸 + 毫针 + 干针 + 火针 + 激光穴位照射 + 穴位贴敷 + 针刺治疗 + 针灸疗法 + 激光穴位 + 穴位注射 + 艾灸 + 灸法 + 灸疗 + 灸术 + 腹针 + 脐针 + 穴位埋线 + 耳针 + 耳针疗法 + 耳穴疗法 + 头皮针 + 头针疗法 + 头针 + 穴位按压 + 埋针 + 皮内针 + 经皮穴位电刺激)) AND ((SU=经前期综合征 + 经前期紧张 + 月经前焦虑障碍 + 经前期紧张症 + 经前期紧张综合症 + 经前烦躁障碍症 + 经前烦躁不安症 + 经前期烦躁障碍) or TI= (经前期综合征 + 经前期紧张 + 月经前焦虑障碍 + 经前期紧张症 + 经前期紧张综合症 + 经前烦躁障碍症 + 经前烦躁不安症 + 经前期烦躁障碍) or KY= (经前期综合征 + 经前期紧张 + 月经前焦虑障碍 + 经前期紧张症 + 经前期紧张综合症 + 经前烦躁障碍症 + 经前烦躁不安症 + 经前期烦躁障碍) OR AB= (经前期综合征 + 经前期紧张 + 月经前焦虑障碍 + 经前期紧张症 + 经前期紧张综合症 + 经前烦躁障碍症 + 经前烦躁不安症 + 经前期烦躁障碍))  **133**

**VIP**

**Date Run: March 18, 2025**

**Search Details ：**

(U=(“针刺” or “电针” or “针灸” or “激光针” or “体针” or “温针灸” or “毫针” or “干针” or “火针” or “激光穴位照射” or “穴位贴敷” or “针刺治疗” or “针灸疗法” or “激光穴位” or “穴位注射” or “艾灸” or “灸法” or “灸疗” or “灸术” or “腹针” or “脐针” or “穴位埋线” or “耳针” or “耳针疗法” or “耳穴疗法” or “头皮针” or “头针疗法” or “头针” or “穴位按压” or “埋针” or “皮内针” or “经皮穴位电刺激”)) AND (U=(“经前期综合征” or “经前期紧张” or “月经前焦虑障碍” or “经前期紧张症” or “经前期紧张综合症” or “经前烦躁障碍症” or “经前烦躁不安症” or “经前期烦躁障碍”))  **79**

**Wanfang**

**Date Run: March 18, 2025**

**Search Details ：**

全部:(“针刺” or “电针” or “针灸” or “激光针” or “体针” or “温针灸” or “毫针” or “干针” or “火针” or “激光穴位照射” or “穴位贴敷” or “针刺治疗” or “针灸疗法” or “激光穴位” or “穴位注射” or “艾灸” or “灸法” or “灸疗” or “灸术” or “腹针” or “脐针” or “穴位埋线” or “耳针” or “耳针疗法” or “耳穴疗法” or “头皮针” or “头针疗法” or “头针” or “穴位按压” or “埋针” or “皮内针” or “经皮穴位电刺激”) and 全部:(“经前期综合征” or “经前期紧张” or “月经前焦虑障碍” or “经前期紧张症” or “经前期紧张综合症” or “经前烦躁障碍症” or “经前烦躁不安症” or “经前期烦躁障碍”) 1**30**

**China Biomedical Literature Database (CBM)**

**Date Run: March 18, 2025**

**Search Details ：**

( "针刺"[全部字段:智能] OR "电针"[全部字段:智能] OR "针灸"[全部字段:智能] OR "激光针"[全部字段:智能] OR "体针"[全部字段:智能] OR "温针灸"[全部字段:智能] OR "毫针"[全部字段:智能] OR "干针"[全部字段:智能] OR "火针"[全部字段:智能] OR "激光穴位照射"[全部字段:智能] OR "穴位贴敷"[全部字段:智能] OR "针刺治疗"[全部字段:智能] OR "针灸疗法"[全部字段:智能] OR "激光穴位"[全部字段:智能] OR "穴位注射"[全部字段:智能] OR "艾灸"[全部字段:智能] OR "灸法"[全部字段:智能] OR "灸疗"[全部字段:智能] OR "灸术"[全部字段:智能] OR "腹针"[全部字段:智能] OR "脐针"[全部字段:智能] OR "穴位埋线"[全部字段:智能] OR "耳针"[全部字段:智能] OR "耳针疗法"[全部字段:智能] OR "耳穴疗法"[全部字段:智能] OR "头皮针"[全部字段:智能] OR "头针疗法"[全部字段:智能] OR "头针"[全部字段:智能] OR "穴位按压"[全部字段:智能] OR "埋针"[全部字段:智能] OR "皮内针"[全部字段:智能] OR "经皮穴位电刺激"[全部字段:智能]) AND( "经前期综合征"[常用字段:智能] OR "经前期紧张"[常用字段:智能] OR "月经前焦虑障碍"[常用字段:智能] OR "经前期紧张症"[常用字段:智能] OR "经前期紧张综合症"[常用字段:智能] OR "经前烦躁障碍症"[常用字段:智能] OR "经前烦躁不安症"[常用字段:智能] OR "经前期烦躁障碍"[常用字段:智能]) **91**

**Supplementary Methods 2 –** Conversion formula for standard deviation (SD).

**Standard Error to Standard Deviation:**

SD=SE×$\sqrt{N}$ where SE is the standard error and N is the sample size.

**95% Confidence Interval to Standard Deviation:**

**1** If the sample size of the test and control groups is greater than or equal to 100: SD=$\sqrt{N}$×(Upper limit of credible intervals - Lower limit of credible intervals)/3.92

**2** If the sample size of the test and control groups is less than or equal to 60: SD=$\sqrt{N}$×(Upper limit of credible intervals - Lower limit of credible intervals)/ tinv (1-0.95,n-1)

**3** For studies with sample sizes between 60 and 100 in each group, both of the above methods can be used.

**Range** **converted SD:**

SD= (Upper limit - lower limit)/4

**Quartile** **converted SD:**

SD= (Upper limit - lower limit)/1.35

Tinv: Represent probabilities, degrees of freedom in excel sheet**Supplementary Methods 3 –** Explanation of technical terms.

| **Standardized mean difference (SMD)** | **Standardized Mean Difference (SMD)** is a statistical measure used to quantify the effect size between two groups. It standardizes the difference between means to allow for comparison across studies with different scales or units. This standardization is achieved by dividing the mean difference by the pooled standard deviation. |
| --- | --- |
| **95% credible intervals (CrI)** | **95% Credible Intervals (CrI)** are used in Bayesian statistics to represent the uncertainty of a parameter estimate. They indicate the range within which the parameter value is believed to lie with 95% probability, given the Bayesian posterior distribution. Unlike frequentist confidence intervals, credible intervals provide a direct probability statement about the parameter values based on the data and prior information. Specifically, a 95% credible interval means that there is a 95% probability that the parameter falls within this range. The interval is calculated using the quantiles of the posterior distribution, offering a direct probabilistic interpretation of the parameter estimate. |
| **Statistical difference** | **Statistical difference** refers to the difference observed between two or more samples, groups, or conditions that is determined to be unlikely due to random variation alone, based on statistical testing. Specifically, statistical difference is established through hypothesis testing (such as t-tests, ANOVA, etc.) to assess whether the observed differences are significant. If the p-value is below a predetermined significance level (e.g., 0.05), the difference is considered statistically significant, meaning that the difference is likely to be a true effect rather than a result of random fluctuation. Statistical difference emphasizes systematic effects in the data rather than random variability. |
| **Heterogeneity** | **Heterogeneity** refers to the variability or differences observed in samples, data, or effects within statistical analysis or research. Specifically, heterogeneity can be categorized into several types: Study Heterogeneity, Statistical Heterogeneity and Clinical Heterogeneity. Identifying and assessing heterogeneity is crucial for understanding the generalizability and practical application of research findings. Researchers may use methods such as subgroup analysis, sensitivity analysis, or random-effects models to better understand and interpret the variability in effects. |
| **Transitivity assumption** | **Transitivity assumption** is a key concept in network meta-analysis used to infer and compare the effects of multiple treatments. The core of the transitivity assumption is that if the effect of Treatment A versus Treatment B is known, and the effect of Treatment B versus Treatment C is known, then the effect of Treatment A versus Treatment C can be inferred. This means that comparisons and inferences between treatment options can be made even in the absence of direct head-to-head comparison data. Specifically, the transitivity assumption includes the following aspects: Consistency, Similarity and Absence of Bias. The validity of the transitivity assumption is crucial for the results and conclusions of network meta-analysis. If the transitivity assumption is not met, it may lead to inaccurate estimates of treatment effects, thus necessitating careful testing and validation of this assumption during network meta-analysis. |

**Supplementary Methods 4 –** Inclusion in the list of studies.

1. Yuan C F, Li M. Randomized Controlled Trial of Umbilical Moxibustion with Getting through Ren Meridian on Premenstrual Syndrome of Liver Depression and Kidney Deficiency[J]. World Latest Medicine Information (Electronic Version),2021,21(25):229-230.

1. Huang X. Analysis of the effect of moxibustion umbilical cord and Tong Ren method in the treatment of liver depression and kidney deficiency type premenstrual syndrome[J]. Inner Mongolia Journal of Traditional Chinese Medicine,2024,43(03):99-101.
2. Lu M X. Research on the method of acupuncture for liver and qi regulation[J]. Journal of Shandong University of TCM,1999,(01):31-34+79.
3. Gu A H. Summary of 30 cases of premenstrual tension syndrome treated by acupuncture with the combination of eight methods of Ling Gui and identification of acupuncture points[J]. Hunan Journal of Traditional Chinese Medicine,2008,(05):30-31.
4. Xie H L, Huang S Z, Liu Y F ,et al. Clinical Observation of Needle Method of Spleen Fortifying and Liver Soothing for Patients with PMS with Liver－Qi Invation[J]. ACTA Chinese Medicine and Pharmacology,2010,38(02):73-75.
5. Song Y , Li S Y, He J L, et al. Clinical observation on 40 cases of auricular acupoint pressure with self-care in the treatment of mild and moderate premenstrual syndrome with liver qi reversal[J]. Jou- rnal of New Chinese Medicine,2014,46(12):186-188.
6. Simsek Kucukkelepce D, Unver H, Nacar G, et al. The effects of acupressure and yoga for coping with premenstrual syndromes on premenstrual symptoms and quality of life[J]. Complementary Therapies in Clinical Practice, 2021, 42: 101282.
7. Shin KR, Ha JY, Park HJ, et al. The effect of hand acupuncture therapy and hand moxibustion therapy on premenstrual syndrome among Korean women[J]. West J Nurs Res,2009,31(2):171-186.
8. Mirghafourvand M, Abdolalipour S, Mohamadi Bolbanabad A, et al. Comparison of the effect of teaching coping skills and acupressure on premenstrual stress: a randomized controlled trial[J]. Discov Ment Health,2025,5(1):31.
9. Korelo RIG, Moreira NB, Miguel BAC, et al. Effects of Auriculotherapy on treatment of women with premenstrual syndrome symptoms: A randomized, placebo-controlled clinical trial[J]. Complement Ther Med, 2022,66,102816.
10. Habek D, Habek JC, Barbir A. Using acupuncture to treat premenstrual syndrome[J]. Arch Gynecol Obstet, 2002,267(1):23-26.
11. Liu X Y, Han N. Observation on therapeutic effect of acupoint catgut embedding therapy on premenstrual syndrome[J]. Chinese Acupuncture and Moxibustion,2006,(04):265-266.
12. Fan X T, Shen X S, Lin C Y, et al. Optimization Scheme of Zhuang Acupuncture Therapy Treating Premenstrual Syndrome[J]. Acupuncture Clinical Journal,2018,34(10):39-42.
13. Lu H L, Zhu Y, Yu X H, et al. Clinical observation of auricular acupressure combined with mild moxibustion in treatment of premenstrual syndrome with liver qi stagnation and spleen deficiency[J]. Guangxi Medical Journal,2022,44(06):581-584+595.
14. Lu H L, Zhu Y, Yu X H, et al. Clinical observation of auricular acupressure combined with mild moxibustion in treatment of premenstrual syndrome with liver qi stagnation and spleen deficiency[J]. Guangxi Medical Journal,2022,44(06):581-584+595.
15. Zhi L X. Random Comparison on the Therapeutic Effects of Hypodermic Catgut Embedding to Treat Premenstrual Syndrome with Invasion of the Hyperactive Liver-qi[J]. Sichuan Traditional Chinese Medicine,2007,(12):111-114.
16. Hong Y F. Clinical Therapeutic effect of Scalp Acupuncture on Premenstrual Tension Syndrome[J]. Chinese Acupuncture and Moxibustion,2002,(09):22-23.
17. Xu Y Y, Sun Y Z. Observations on the efficacy of lumbar dorsal translucency in the treatment of premenstrual syndrome[J]. Acupuncture and Moxibustion Clinical Journal,2006,(05):37-38.
18. Xu T S. Clinical therapeutic effect of point-injection combined with body acupuncture on premenstrual tention syndrome[J]. Chinese Acupuncture and Moxibustion,2005,(04):253-254.
19. Guo S Y, Sun Y Z. Comparison Between Acupuncture and Medication in Treatment of Premenstrual Syndrome[J]. Shanghai Acupuncture and Moxibustion Journal,2004,(01):5-6.
20. Jiang W, Li Y, Sun J. Clinical Study on Treatment of Premenstrual Tension Syndrome with Auricular Point Sticking[J]. Chinese Acupuncture and Moxibustion,2002,(03):165-167.

**Supplementary Table S1 –** Study characteristics of the included studies.

| **ID** | **Study** | **Design** | **Participants** | | **Interventions charateristics** | | **Outcome** |
| --- | --- | --- | --- | --- | --- | --- | --- |
|  |  |  | **N** | **Age** | **Duration and frequency of interventions** | c**lass** |  |
| **1** | Chongfen Yuan  2021 | RCT | 30 | 28.4±4.2 | Umbilical moxibustion，1 time/week, 1.5h each time, total treatment 3 menstrual cycles | Moxibustion | Overall effective rate |
|  |  |  | 30 | 28.1±3.9 | Treatment 1 time /d, needle retention 30min, each menstrual cycle will receive 5-7 treatments, a total of 3 menstrual cycles | Acupuncture |  |
| **2** | Xin Huang  2024 | RCT | 36 | 28.43±4.24 | Umbilical moxibustion，1 time/week, 1.5h each time, total treatment 3 menstrual cycles | Moxibustion | Overall effective rate |
|  |  |  | 36 | 28.46±4.16 | Treatment 1 time /d, needle retention 30min, each menstrual cycle will receive 5-7 treatments, a total of 3 menstrual cycles | Acupuncture |  |
| **3** | Mingxia Lu  1995 | RCT | 26 | 33.8±5.0 | The treatment was carried out once a day, leaving the needle in place for 20 min, starting on the 16th day of the menstrual cycle, until the onset of menstruation as a course of treatment, and the efficacy was counted after 2 consecutive courses of treatment. | Acupuncture | Overall effective rate； symptom and sign scores |
|  |  |  | 30 | 32.9±5.9 | Non-intervention | Blank Control Group |  |
| **4** | Anhua Gu  2008 | RCT | 30 | NA | Treatment leave the needle for 30min, every 10min line needle 1 time, starting from 10 days before each menstruation, once a day, after the onset of menstruation to stop the treatment, a total of 3 menstrual cycles of treatment | Linggui-Bafa acupuncture | Overall effective rate |
|  |  |  | 30 | NA | Treatment leave the needle for 30min, every 10min line needle 1 time, starting from 10 days before each menstruation, once a day, after the onset of menstruation to stop the treatment, a total of 3 menstrual cycles of treatment | Acupuncture |  |
| **5** | Hongliang Xie  2010 | RCT | 20 | NA | Start treatment 14 days before the onset of menstruation, once a day until the end of the 2nd menstrual cycle, and observe for 3 more menstrual cycles after the end of treatment | Jianpi-Shugan Acupuncture | Overall effective rate |
|  |  |  | 20 | NA | Start treatment 14 days before the onset of menstruation, once a day until the end of the 2nd menstrual cycle, and observe for 3 more menstrual cycles after the end of treatment | Acupuncture |  |
| **6** | Yang Song  2014 | RCT | 40 | 26.40±2.88 | Auricular acupressure therapy was added to the self-care regimen and was applied 2 weeks before the subject's menstrual period and continued for 3 menstrual cycles | Ear Acupressure | Overall effective rate； symptom and sign scores |
|  |  |  | 40 | 28.72±2.97 | Instructing the subjects to adopt methods of self-care such as dietary modification, emotional relief, reasonable rest and relaxation, and appropriate exercise | Placebo Control Group |  |
| **7** | Didem  2021 | RCT | 51 | NA | To provide circulation before the pressure, a 30-min massage was given to each acupressure point, which was followed by consecutive pressures applied for 90s. Twice a week and 24 times in total | Acupressure | Symptom and sign scores |
|  |  |  | 54 | NA | Non-intervention | Blank Control Group |  |
| **8** | Kyung Rim Shin  2009 | RCT | 7 | NA | 1 treatment every 3 days for 15 min, 10 treatments in total | Acupuncture | Symptom and sign scores |
|  |  |  | 8 | NA | 1 treatment every 3 days for 30 min, 10 treatments in total | Moxibustion |  |
|  |  |  | 7 | NA | Non-intervention | Blank Control Group |  |
| **9** | Mojgan Mirghafourvand  2025 | RCT | 30 | 22.3±2.9 | 60s twice a day for 3 menstrual cycles | Acupressure | Symptom and sign scores |
|  |  |  | 30 | 22.8±2.3 | Non-intervention | Blank Control Group |  |
| **10** | R. I. G. Korelo  2022 | RCT | 30 | 21.2±3.1 | Treatment once a week, starting 5 days before menstruation until the second day of menstruation for 8 weeks | Auricular Microneedle | Symptom and sign scores |
|  |  |  | 30 | 20.5±1.8 | Non-intervention | Blank Control Group |  |
| **11** | Dubravko Habek  2002 | RCT | 18 | 30.6±8.4 | Treatments were conducted during the third luteal phase of the menstrual cycle, every second day (7 days) for a period of 30 min a day | Acupuncture | Symptom and sign scores |
|  |  |  | 17 | 29.8±7.3 | Treatments were conducted during the third luteal phase of the menstrual cycle, every second day (7 days) for a period of 30 min a day | Placebo Control Group |  |
| **12** | Xiangyang Liu  2006 | RCT | 44 | NA | 1 treatment 15 days before menstruation for 3 consecutive menstrual periods | Hypodermic Catgut Embedding | Overall effective rate |
|  |  |  | 44 | NA | Take 20mg per dose once daily in the morning for 3 months | Conventional Western Medicine Group |  |
| **13** | Xiaoting Fan  2018 | RCT | 24 | 28.5±7.75 | Start the treatment at the time of menstrual cleansing, stop the treatment at the onset of menstruation, treat every 2 days, leave the needle for 30 min each time, for a total of 3 menstrual cycles | “8”shaped ring acupuncture method | Overall effective rate； symptom and sign scores |
|  |  |  | 24 | 29.2±6.71 | Start on day 11 of the menstrual cycle and stop on day 25 of the menstrual cycle for 3 consecutive menstrual cycles | Conventional Western Medicine Group |  |
| **14** | Huiling Lu  2022 | RCT | 30 | 24.27±2.64 | 30min each time, starting from the 20th day of the menstrual cycle until the onset of menstruation, for a total of 3 menstrual cycles | Ear Acupressure Combined with Moxibustion | Overall effective rate； symptom and sign scores |
|  |  |  | 30 | 23.57±2.08 | Starting on the 20th day of the menstrual cycle, 20 mg/d once a day for 3 consecutive menstrual cycles | Conventional Western Medicine Group |  |
| **15** | Yuanzheng Sun  2004 | RCT | 30 | 30.06±1.93 | After obtaining qi, leave the needle for 30 minutes, during which time the needle is performed once every 5 minutes. Treatment was started once a day, 14 days before menstruation, and stopped during menstruation, for a total of 3 menstrual cycles | Acupuncture | Overall effective rate； symptom and sign scores |
|  |  |  | 31 | 31.02±1.9 | Started 14 days before menstruation, stopped during menstruation, total of 3 menstrual cycles of treatment | Conventional Western Medicine Group |  |
| **16** | Liangxi Zhi  2007 | RCT | 54 | 33.61±5.5 | The first intervention was administered 4 days before the menstrual cycle; subsequent interventions were administered 14 days before each menstrual cycle. The intervention was carried out for 3 consecutive menstrual cycles | Hypodermic Catgut Embedding | Overall effective rate； symptom and sign scores |
|  |  |  | 54 | 33.35±5.3 | The first intervention started oral medication 4 days before the menstrual cycle until the onset of menstruation; thereafter, oral medication was started 14 days before each menstrual cycle until the onset of menstruation. The intervention was carried out for 3 consecutive menstrual cycles | Conventional Western Medicine Group |  |
| **17** | Yufang Hong  2002 | RCT | 35 | NA | Leave the needle in for 1 hour, 3 times a week, 5 days rest after menstruation, total treatment for 3 menstrual cycles | Scalp Acupuncture Combined with electroacupuncture | Overall effective rate |
|  |  |  | 31 | NA | Start taking the drug 14 days before menstruation and stop taking the drug at the end of menstrual period, total treatment for 3 menstrual cycles | Conventional Western Medicine Group |  |
| **18** | Yingying Xu  2006 | RCT | 30 | 30.97±4.91 | Leave the needle in for 30 min, once a day, start the treatment 14 days before menstruation, stop the treatment during menstruation, and treat for 3 consecutive menstrual cycles | Acupuncture | Overall effective rate |
|  |  |  | 30 | 31.20±3.97 | 6mg orally daily for 10 days starting on day 16 of the menstrual cycle for 3 menstrual cycles | Conventional Western Medicine Group |  |
| **19** | Tianshu Xu  2005 | RCT | 54 | NA | Beginning 10 days before each menstrual period, treatment every 3 days, stop treatment after the onset of menstruation, for a total of 3 menstrual cycles | Acupuncture Combined with Point Injection | Overall effective rate |
|  |  |  | 48 | NA | Start 10 days before menstruation, stop taking at the onset of menstruation, for a total of 3 menstrual cycles | Conventional Western Medicine Group |  |
| **20** | Yingshu Guo  2004 | RCT | 35 | 30.73±4.00 | Leave the needle for 30min, treatment once a day, start treatment 14 days before menstruation, stop during menstruation, treatment for a total of three menstrual cycles | Acupuncture | Overall effective rate； symptom and sign scores |
|  |  |  | 31 | 31.02±4.25 | Started 14 days before menstruation, stopped at the end of menstruation, for a total of 3 menstrual cycles | Conventional Western Medicine Group |  |
| **21** | Wen Jiang  2002 | RCT | 32 | 30.23±7.30 | Allow patients to press each point by themselves 6 times a day, 6 times each time, alternately every 3 days or so, starting on the 20th day of the menstrual cycle, until the onset of menstruation, for 3 consecutive menstrual cycles of treatment | Ear Acupressure | Overall effective rate |
|  |  |  | 16 | 30.17±8.30 | 3 times daily, starting on the 20th day of the menstrual cycle, until the onset of menstruation, for 3 consecutive menstrual cycles | Conventional Western Medicine Group |  |

**Supplementary Figure S1 –** Excluded studies.


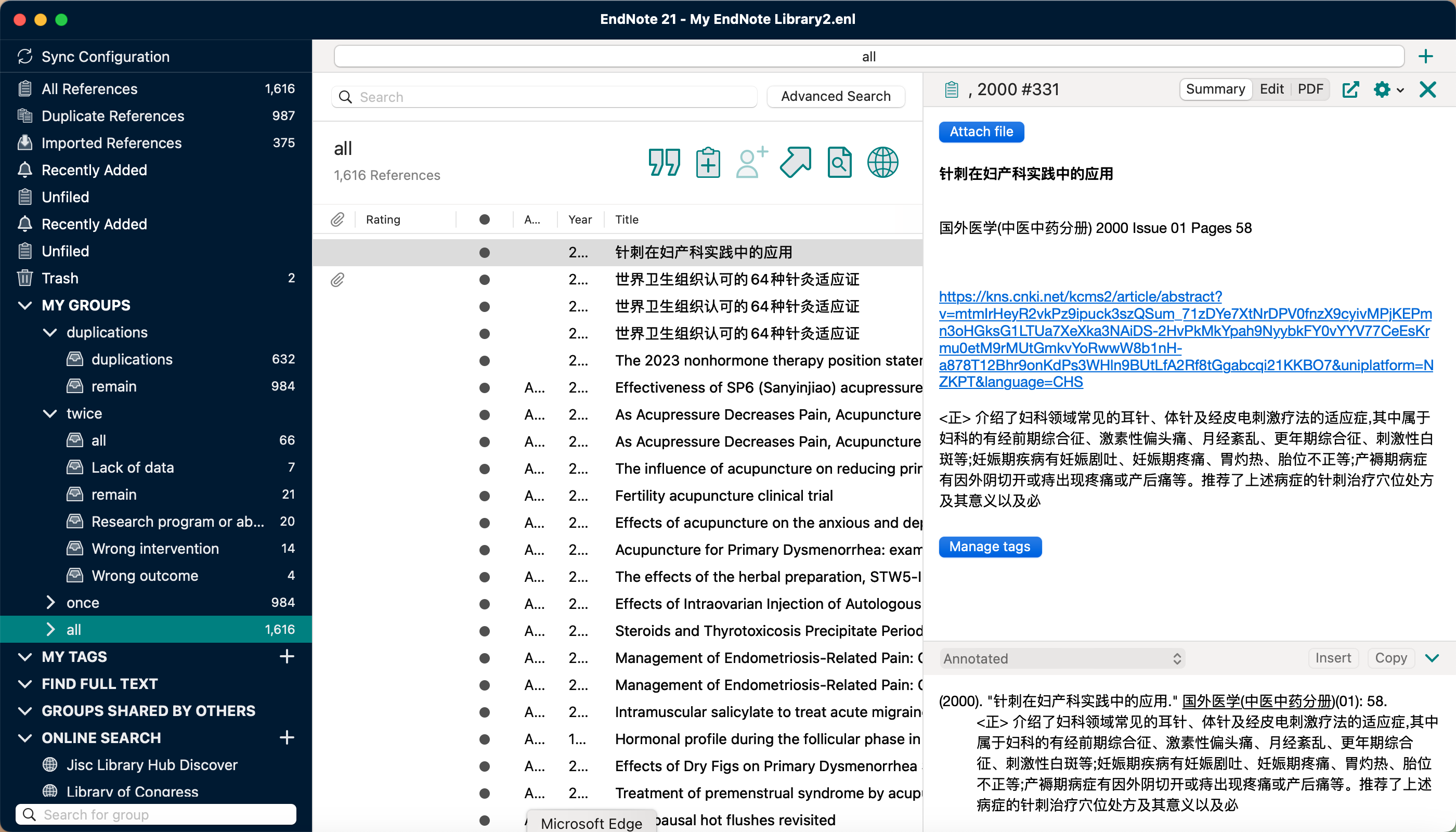


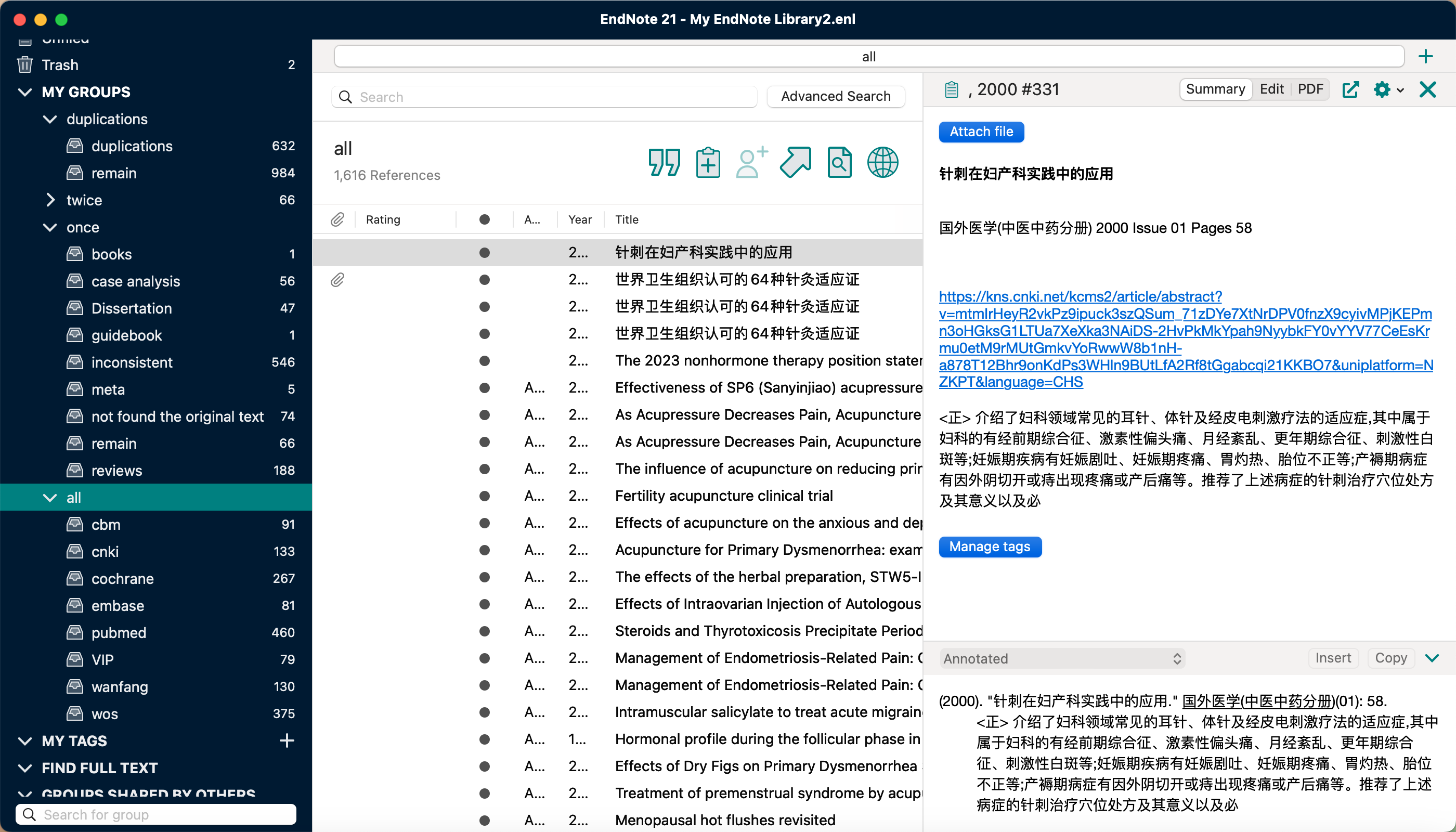


**Supplementary Table S2 –** Risk of bias table of included studies.

| **Study** | **Randomization process** | **Deviations from intended interventions** | **Missing outcome data** | **Measurement of the outcome** | **Selection of the reported result** | **Overall risk of bias** |
| --- | --- | --- | --- | --- | --- | --- |
| 1. Chongfen Yuan, 2021 | High | Unclear | Unclear | Low | Unclear | High |
| 1. Xin Huang, 2024 | High | Unclear | Unclear | Low | Unclear | High |
| 1. Mingxia Lu, 1995 | Unclear | Unclear | Unclear | Low | Unclear | Unclear |
| 1. Anhua Gu, 2008 | Unclear | Unclear | Unclear | Low | Unclear | Unclear |
| 1. Hongliang Xie, 2010 | Unclear | Unclear | Unclear | Low | Unclear | Unclear |
| 1. Yang Song, 2014 | Low | Unclear | Unclear | Low | Unclear | Unclear |
| 1. Didem, 2021 | Low | Low | Low | Low | Unclear | Low |
| 1. Kyung Rim Shin, 2009 | Unclear | Unclear | Unclear | Low | Unclear | Unclear |
| 1. Mojgan Mirghafourvand, 2025 | Low | Low | Low | Low | Unclear | Low |
| 1. R. I. G. Korelo, 2022 | Unclear | Low | Low | Low | Unclear | Low |
| 1. Dubravko Habek, 2002 | Unclear | Unclear | Unclear | Low | Unclear | Unclear |
| 1. Xiangyang Liu, 2006 | Low | Unclear | Unclear | Low | Unclear | Unclear |
| 1. Xiaoting Fan, 2018 | Low | Unclear | Unclear | Low | Unclear | Unclear |
| 1. Huiling Lu, 2022 | Low | Unclear | Unclear | Low | Unclear | Unclear |
| 1. Yuanzheng Sun, 2004 | High | Unclear | Unclear | Low | Unclear | High |
| 1. Liangxi Zhi, 2007 | Unclear | Unclear | Low | Low | Unclear | Unclear |
| 1. Yufang Hong, 2002 | High | Unclear | Unclear | Low | Unclear | High |
| 1. Yingying Xu, 2006 | High | Unclear | Unclear | Low | Unclear | High |
| 1. Tianshu Xu, 2005 | Low | Unclear | Unclear | Low | Unclear | Unclear |
| 1. Yingshu Guo, 2004 | Unclear | Unclear | Unclear | Low | Unclear | Unclear |
| 1. Wen Jiang, 2022 | High | Unclear | Unclear | Low | Unclear | High |

**Supplementary Figure S2–** The results of the Begg test for overall effective rate.


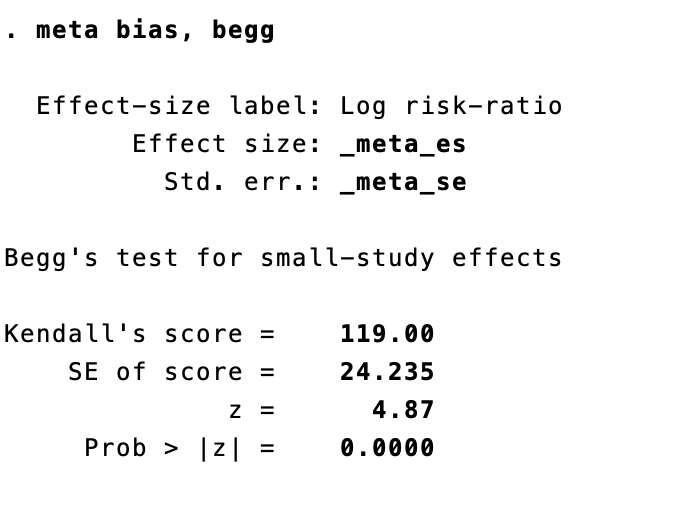


**Supplementary Figure S3–** The results of the Begg test for symptom and sign scores.


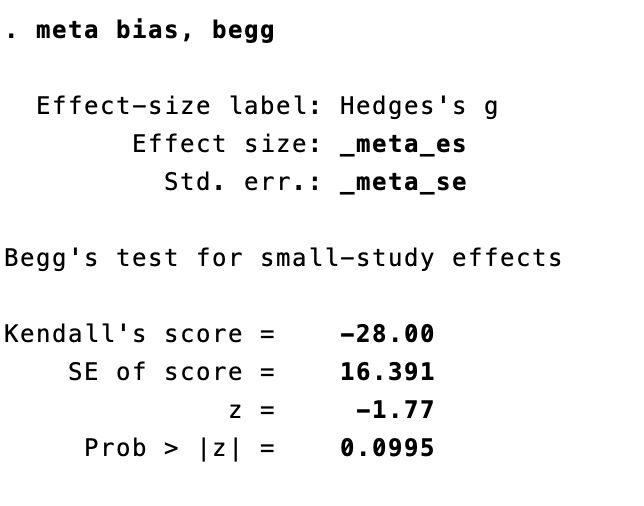


**Supplementary Table S3**–List of Abbreviations.

| Number | words and phrases | abbreviations |
| --- | --- | --- |
| 1 | Premenstrual syndrome | PMS |
| 2 | Traditional Chinese Medicine | TCM |
| 3 | Randomized controlled trial | RCT |
| 4 | Premenstrual Dysphoric Disorder | PMDD |
| 5 | Oral Contraceptive Pill | OCP |
| 6 | Selective Serotonin Reuptake Inhibitor | SSRI |
| 7 | Gonadotropin-Releasing Hormone Agonists | GnRH-a |
| 8 | Complementary and Alternative Medicine | CAM |
| 9 | Sanyinjiao | SP6 |
| 10 | Taichong | LR3 |
| 11 | Guanyuan | RN4 |
| 12 | Network meta-analyse | NMA |
| 13 | Chinese National Knowledge Infrastructure | CNKI |
| 14 | China Biomedical Medicine Database | CBM |
| 15 | Standard deviation | SD |
| 16 | Confidence interval | CI |
| 17 | Mean difference | MD |
| 18 | I-square | I² |
| 19 | Functional connectivity | FC |
